# Non-marine palaeoenvironment associated to the earliest tetrapod tracks

**DOI:** 10.1038/s41598-018-19220-5

**Published:** 2018-01-18

**Authors:** Martin Qvarnström, Piotr Szrek, Per E. Ahlberg, Grzegorz Niedźwiedzki

**Affiliations:** 1 Subdepartment of Evolution and Development, Department of Organismal Biology, Evolutionary Biology Centre, Uppsala University, Norbyvägen 18A, 752 36 Uppsala, Sweden; 2 Polish Geological Institute-National Research Institute, Rakowiecka 4 Street, 00-075 Warszawa, Poland

**Keywords:** Palaeontology, Palaeontology

## Abstract

Opinions differ on whether the evolution of tetrapods (limbed vertebrates) from lobe-finned fishes was directly linked to terrestrialization. The earliest known tetrapod fossils, from the Middle Devonian (approximately 390 million years old) of Zachełmie Quarry in Poland, are trackways made by limbs with digits; they document a direct environmental association and thus have the potential to help answer this question. However, the tetrapod identity of the tracks has recently been challenged, despite their well-preserved morphology, on account of their great age and supposedly shallow marine (intertidal or lagoonal) depositional environment. Here we present a new palaeoenvironmental interpretation of the track-bearing interval from Zachełmie, showing that it represents a succession of ephemeral lakes with a restricted and non-marine biota, rather than a marginal marine environment as originally thought. This context suggests that the trackmaker was capable of terrestrial locomotion, consistent with the appendage morphology recorded by the footprints, and thus provides additional support for a tetrapod identification.

## Introduction

The evolution and terrestrialization of tetrapods, beginning during the Middle Devonian period, was one of the most important events in the evolution of vertebrates^1–10^. There has been a major expansion in our understanding regarding this transition during the last three decades, in contrast to the major part of the 20^th^ century when the iconic *Ichthyostega* and *Acanthostega* were the only known Devonian taxa^3,4^. Many new Late Devonian tetrapods have been described, and remains have been found world-wide in Asia^5^, Russia^6^, Europe^7,8^, North America^9^ and Australia^10^. It should be noted that the term “tetrapod” is used throughout this paper to denote a vertebrate with limbs rather than paired fins. The clade of limbed vertebrates comprises the tetrapod crown group plus the most crownward part of the stem group. Successively less crownward parts of the stem group contain the transitional “elpistostegids” *Elpistostege*, *Tiktaalik* and *Panderichthys*, and the more conventionally fish-like “osteolepiforms” such as *Eusthenopteron* and *Gogonasus*^8,11,12^. All currently known Devonian tetrapods are unambiguous members of the stem group; the earliest crown-group tetrapods are of Early Carboniferous age.

**Figure 1:**
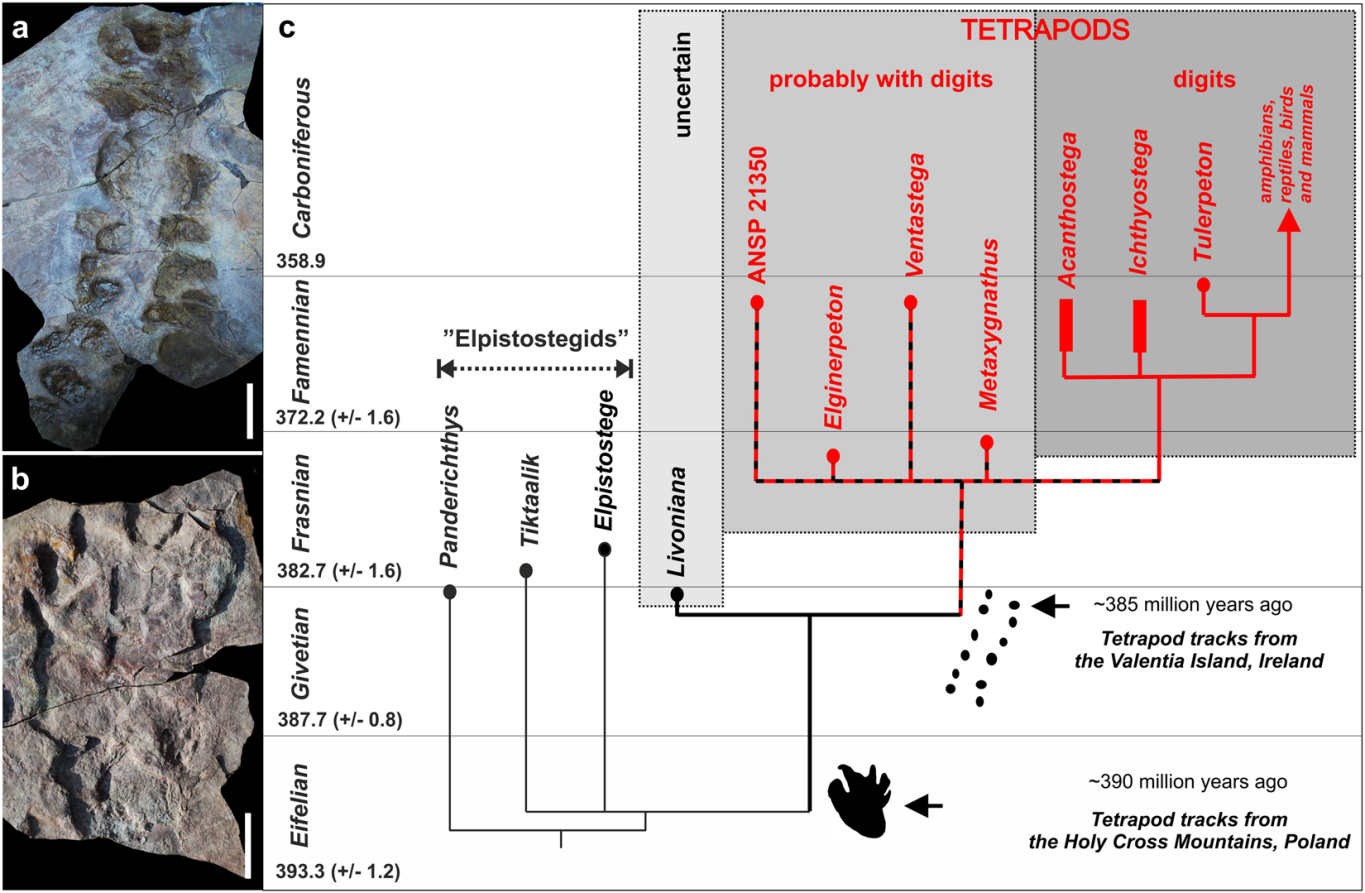
Examples of Middle Devonian tetrapod traces from Zachełmie and phylogenetic implications of these finds. (**a**) Trackway specimen (Muz. PGI 1728.II.7, Geological Museum of the Polish Geological Institute-National Research Institute) showing numerous, in part overlapping prints, presumed direction of locomotion is from bottom to top. (**b**) Large manus or pes imprint with digit impressions (Muz. PGI 1728.II.1). (**c**) Effect of adding the Zachełmie record to the phylogeny of early tetrapods (modified from Niedźwiedzki *et al*.^1^; chronostratigraphic and numerical dates after International Commission on Stratigraphy^16^).

The earliest evidence for tetrapods in the fossil record consists of early Middle Devonian trace fossils (tracks and trackways) from Zachełmie Quarry, in the Holy Cross Mountains (Góry Świętokrzyskie) of south-central Poland^1^. Some of the abundant footprints in the locality are preserved with digit impressions (Fig. 1a,b). The digit imprints, together with the absence of body drags in association with the unambiguously quadrupedal trackways, firmly imply an early tetrapod track maker and disqualify alternative interpretations such as the structures being elpistostegid tracks or fish resting traces with fecal pellets^13^.

It is a remarkable fact that both the earliest (Zachełmie) and the second earliest (Valentia Island, Ireland; latest Middle Devonian^14,15^) tetrapod occurrences in the fossil record consist of footprints. The earliest definite tetrapod body fossils come from Late Devonian sediments that are approximately 14 million years younger than the Zachełmie tracks, while the earliest known elpistostegid, *Panderichthys*, is contemporary with the Valentia Island tracks and thus some 5 million years younger than Zachełmie^16,17^. This implies that the evolutionary time frame suggested by the body fossil record, which appears to show elpistostegids being replaced by tetrapods during the Frasnian (early Late Devonian), is an artifact; the elpistostegid-tetrapod split must have occurred prior to the formation of the Zachełmie track-bearing layers^1,2^. Circumstantial evidence in support of this contention is provided by the fragmentary taxon *Livoniana*^18^, which is contemporary with *Panderichthys* but appears to be more derived than any known elpistostegid and could possibly be a very primitive tetrapod.

In addition to their importance for dating the origin of tetrapods, the Zachełmie tracks also provide crucial information about their palaeoecology. Trackways represent traces of living organisms, in contrast to body fossils which most often are subjected to postmortem transportation. As a consequence, the Zachełmie trackways, albeit just representing brief snapshots from the past, have played a prominent role in a number of recent palaeoenvironmental hypotheses that place the emergence of tetrapods in tidal flat environments^1^, flooded woodlands^19^ or shallow-marine lagoons^20,21^.

Here we present a detailed palaeoenvironmental reinterpretation of the trace fossil-bearing section from Zachełmie Quarry, based on a combination of newly collected data and published data from previous studies^1,20,21^. The new data, collected during the last few years from the three tetrapod track-bearing horizons as well as from the general succession in Zachełmie Quarry, demonstrate that the palaeoenvironmental conditions associated with track formation have been misinterpreted^1,19–21^. We propose that the track-bearing interval was deposited in a terrestrial setting in the form of ephemeral lakes that were well separated from marine influences. Our data suggest that the track makers were already capable of effective terrestrial locomotion.

## Results

### Geological background

**Figure 2:**
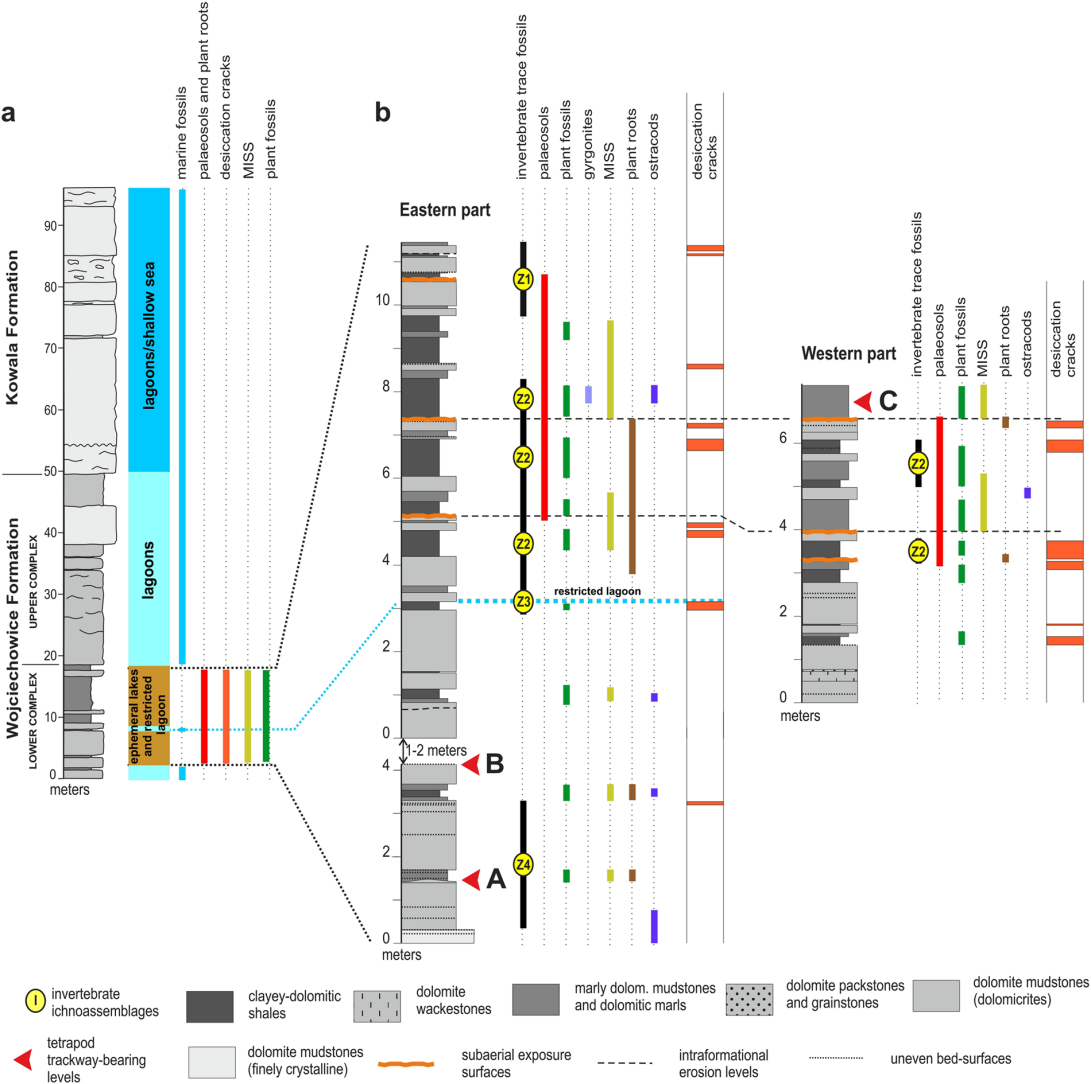
Lithology, stratigraphy and the distribution of fossils in the Zachełmie Quarry section. (**a**) Simplified lithological section with distribution of marine fossils, palaeosols (plant roots), desiccation cracks and MISS (modified from Niedźwiedzki *et al*.^1^ and Narkiewicz *et al*.^21^). (**b**) Enlarged lower part (Lower Complex) showing detailed distribution of lithologies, sedimentary structures, micro- and macrofossils, invertebrate trace fossils, location of tetrapod track-bearing horizons (modified from Narkiewicz *et al*.^21^).

**Figure 3:**
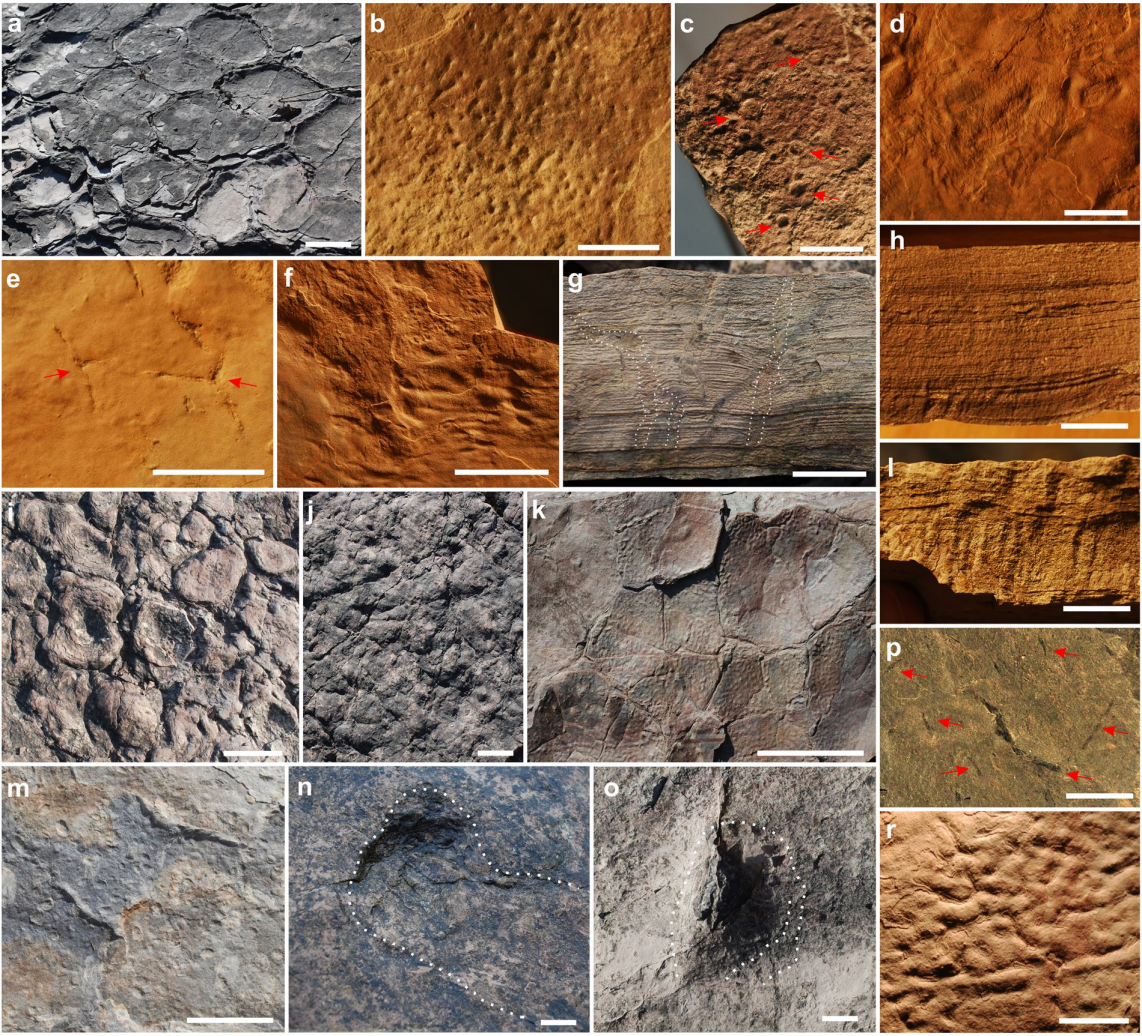
Sedimentary structures, macrofossils, invertebrate trace fossils from the Lower Complex in the Zachełmie Quarry section. (**a**) Surface with desiccation cracks. (**b**) Microbial structures, perhaps representing gas escaping hollows, preserved on microbial mat. (**c**) surface with rain drop structures (red arrows). (**d**) Deformed microbial mat. (**e**) Pseudomorphs after salt crystals. (**f**) Crumpled, deformed microbial mat preserved on clayey dolomitic shale surface. (**g**) Vertical plant roots. (**h**) Clayey dolomitic shale with fine- planar lamination which characterize the microfacies abiotic laminite. (**i**) Surface with stromatolite structures. (**j**) Upper surface of palaeosol horizon. (**k**) Microbial mat with record of desiccation cracks. (**l**) Palaeosol horizon covered by clayey dolomitic shale with abiotic laminite. (**m**) Surface with poorly developed mudcracks and numerous casts after ostracod carapaces and charophyte gyrogonites. (**n, o**) Large arthropod burrows. (**p**) Plant fossils and phytoclasts (red arrows). (**r**) Microbially-induced sedimentary structure. Scale bars in a, i, j, k, 10 cm; scale bar in b, c, d, e, p, 1 cm; scale bar in n, o, 5 cm.

The Devonian dolomitic succession in the locality is composed of a part of the Wojciechowice Formation which is overlain by the lower part of the Kowala Formation^21^. The former is divided by an erosional boundary into a Lower and Upper Complex, which differ remarkably in sedimentological architecture, microfacies and fossil content^21^. All three horizons (A–C) with tetrapod traces are located in the Lower Complex (Fig. 2). The Lower Complex is composed of short shallowing upward cycles (14 or 15 cycles) with laminated dolomite mudstones and contains numerous sedimentary and deformational structures. The Upper Complex, on the other hand, is typified by a more massive bedding and a common presence of dolomite wackestones^21^. The cyclic beds are characterized by a general upward decrease in terrigenous clay admixture and represent a record of repeated gradual shallowing and desiccation^21^. The investigated cyclic beds do not exhibit any signs characteristic of intertidal or supratidal environments^21^. The cycle tops from the Lower Complex are often crowned by surfaces of desiccation cracks and, occasionally, by palaeosols with formation time-spans ranging from hundreds to thousands of years^20^. Brief and extended periods of subaerial exposure are indicated by co-occurrences of desiccation cracks and palaeosols, rain-drop imprints, horizons with vertical plant roots, large arthropod burrows (similar to types described from terrestrial settings) and rare horizons with pseudomorphs after evaporite mineral crystals (Figs 2 and 3). Several horizons with plant-root traces indicate periodically stable plant cover, probably multi-seasonal vegetation.

**Figure 4:**
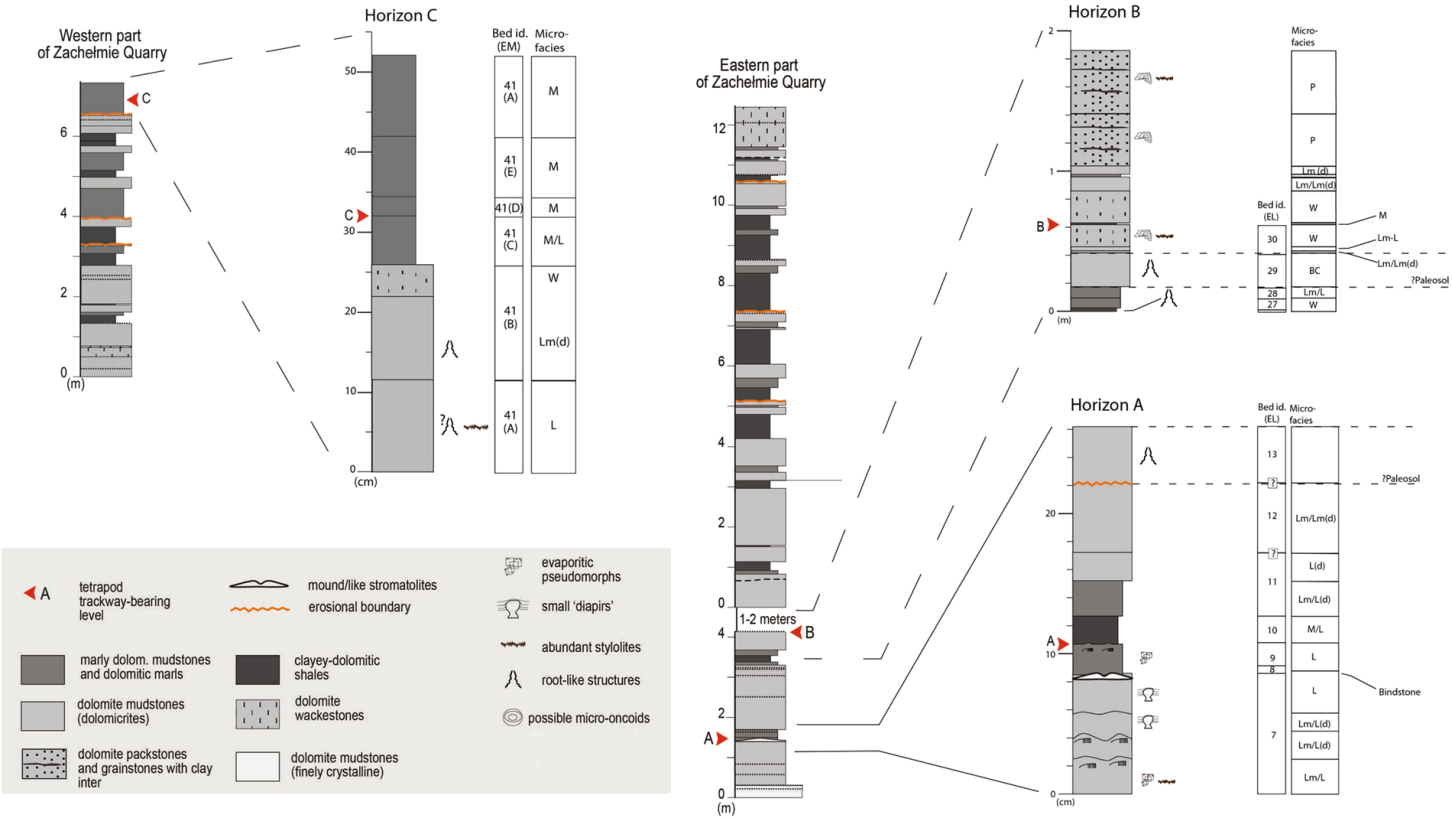
Detailed sedimentary profiles across the three track-bearing horizons (A–C). Bed identification is expressed as a modification of the system of Narkiewicz *et al*.^21^. For bed identification in relation to the track-bearing horizons see Supplementary Information.

The geochemistry of stable isotopes from bulk rock samples from the Lower Complex suggests well-aerated conditions and water temperatures around 30 °C^21^. Both, macro- (e.g., fossilized microbial mats), and microscopic features (e.g., microbial dolomite grains) confirm biologically-induced mineralization during the formation of some laminated dolomite mudstones from the Lower Complex^21^. Overall, nine microfacies types have been described from the Lower Complex^21^ (Fig. 4; see Supplementary Information). For this study, 34 thin sections were produced by specifically targeting the track-bearing horizons and the immediately under/overlying beds. Common microfacies are displayed in Supplementary Information and results across the track-bearing horizons are tabulated, as well as presented as detailed profiles across the track-bearing horizons (Fig. 4) which enables a comprehensive view on the sedimentation during these intervals. From thin-section analyses it is obvious that the track-bearing horizon and the adjacent beds are characterized by clay-rich sediments, a terrigenous input from the closest land. The clay-content varies however and some beds are even classified by their lithology as clayey dolomitic shales.

### Rare earth elements (REEs) data

It was hypothesized that if REEs of bulk rock samples reflect the primary water composition, samples from the Kowala Formation would display marine signatures as the formation marks the initial stages of carbonate platform development. It was furthermore hypothesized that the track-bearing Wojciechowice Formation would show non-marine REE signatures if it was not deposited in a similar marine environment. Furthermore, the eventual presence of cerium anomalies could deduce the prevailing redox conditions during formation of the deposits.

**Figure 5:**
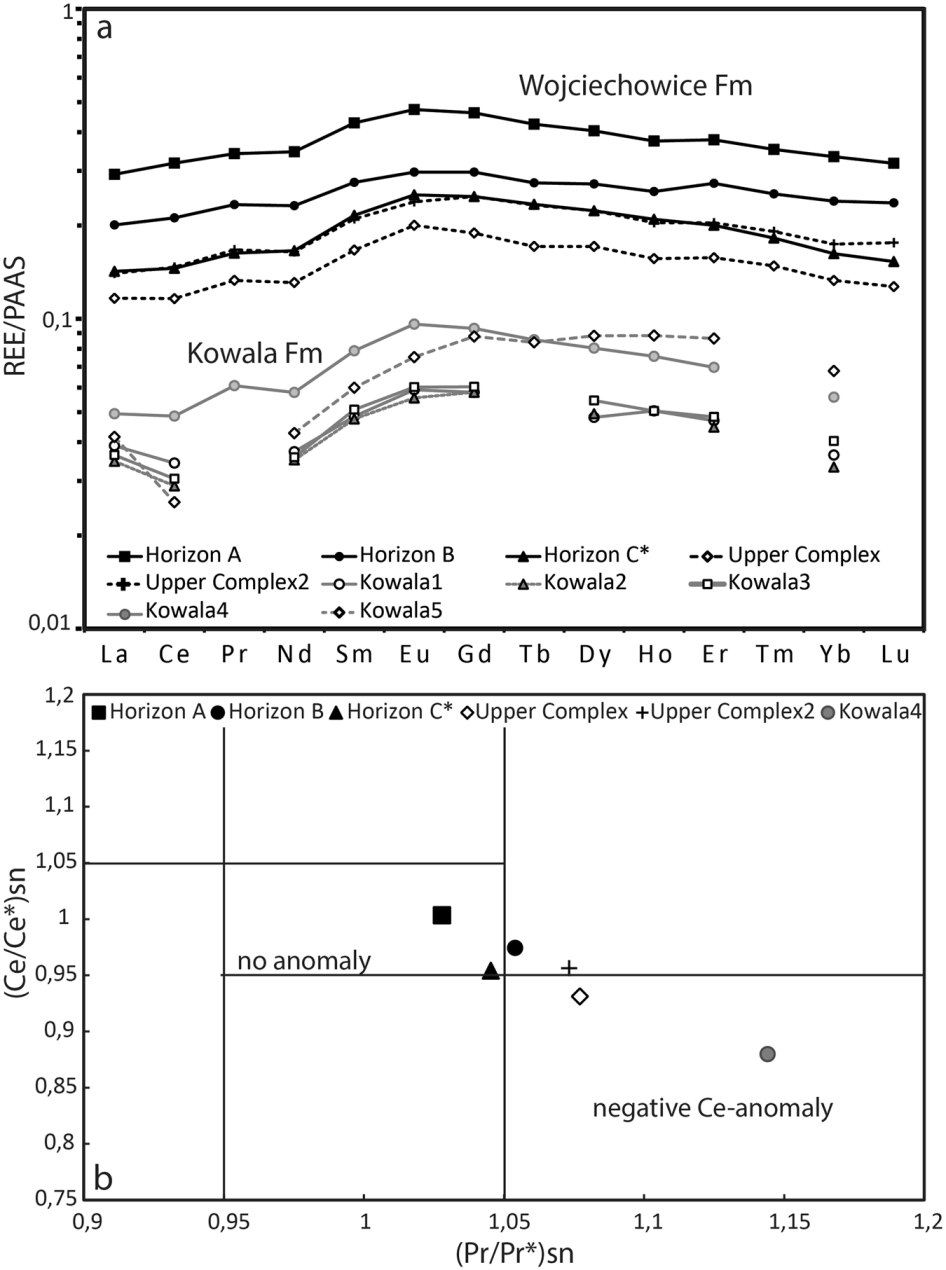
REE data from track-bearing horizons. (**a**) Shale-normalized REE patterns from bulk rock samples of beds from the track-bearing horizons (*marks lateral equivalent), the Upper Complex of the Wojciechowice Formation, and the Kowala Formation. (**b**) Same samples plotted in a graph of calculated (Ce/Ce*)_SN_ vs. (Pr/Pr*)_SN_ values, after Bau & Dulski^38^. Note that only two data points fall into the area characterized by negative cerium anomalies.

The samples from the Wojciechowice Formation, including the track-bearing horizons, display concentrations of REEs about ten times higher than those from the Kowala Formation (Fig. 5a). In addition, the shale-normalized patterns are more flattened, but show a slight bell-shape. One sample from the Upper Complex of the Wojciechowice Formation displays a slight negative cerium anomaly (Fig. 5b), whereas the other samples from the formation display neither positive nor negative cerium anomalies. The measured Praseodymium (Pr) content of the samples from the Kowala Formation is below the detection limit for all samples but one, making it impossible to calculate cerium anomalies for the majority of the samples. The single sample with Pr measures, however, displays a clear negative cerium anomaly (Fig. 5b).

The separation of the two formations based on REE patterns is most likely due to the degree of clay contamination. Clay content has been showed to largely influence REE-measures of bulk rock samples^22,23^, and the amount of individual REEs correlates well with the Al_2_O_3_ content of the samples (e.g., cerium and Al_2_O_3_ contents of the samples display a linear correlation with high confidence, R^2 = ^0.95). The sample from the Wojciechowice Formation with the negative cerium anomaly was also the sample in which the overall REE content was low, and thus the least contaminated by clays. Perhaps this is a remnant of a primary cerium anomaly that precipitated from an oxygenated water body during the deposition of the Upper Complex in general, but this is highly speculative.

The retrieved REE-signatures are not likely a result of, or altered by, fluid interaction during diagenesis. The samples show evidence of a mild diagenetic history, manifested by a low degree of diagenetic recrystallization.

### Tetrapod tracks and trackways

Isolated tracks and trackways were both found *in situ* and on loose slabs from the scree in the Lower Complex part of the section. Two out of the three track-bearing horizons (Horizon A and B) are located on the eastern part of the quarry and one in the western part (Horizon C), (Figs 1 and 4). All track-bearing levels occur in the lower and middle parts of the shallowing upward cycles without evidence of either subaerial exposure or marine influence^21^. Digit marks are variably absent or present depending on the substratum and track-sizes varies as a function of animal size and type of locomotion^1^. In addition to tracks and trackways associated to walking tetrapods, isolated large prints interpreted as being made by swimming tetrapods kicking against the substratum have been described^1^. As in other tracksites from younger late Middle^24^ or Late Devonian^25^ strata there are documented manus and pes imprints, which together with the spacing and stride length of the tracks excludes tetrapod-ancestors such as elpistostegids as trackmakers^1^. The largest tracks are approximately twice the linear dimensions of the best preserved *Ichthyostega* foot skeleton, suggesting that the early tetrapod responsible for these tracks could have been up to 2.5 meter in length^1^. So far, however, no early tetrapod body fossils have been retrieved from the area.

### Body-fossil record

The body-fossil record in the Lower Complex of the Wojciechowice Formation is very poor^21^ and comprises only: conodonts, foraminifers, ostracods and small bivalve remains restricted to narrow stratigraphical intervals situated below and above the track-bearing interval; a few degraded palynomorphs (e.g., spores and acritarchs) gathered from a single sample; structures formed by plant roots; several isolated of fish remains; macroscopic fragments of vascular plants; and charophyte gyrogonites (Fig. 3; see Supplementary Information). The marine body fossils (e.g., conodonts, ostracods, foraminifers) of the Lower Complex interval are generally poorly preserved and clearly fractured from reworking. This suggests episodic local transportation and re-working of the small phosphatic (conodont remains) or shelly fauna from a neighbouring marginal marine environment into the muddy and low-energy depositional environment of the Lower Complex, perhaps in connection with storm events, implying that the general poor body fossil content in the Lower Complex is not just a preservation bias. It should also be added that there is a complete absence of marine body fossils in the interval with tetrapod tracks (Fig. 2). In contrast, the Upper Complex contains numerous layers relatively rich in marine fossils, such as echinoderms (crinoids), bryozoans, scolecodonts, fish and conodont fossils, but lacks horizons with desiccation cracks or palaeosols^21^.

In two samples from the Lower Complex poorly preserved remains of calcitic gyrogonites of charophyte algae have been found (Fig. 3). These observations are still preliminary and will be covered more thoroughly in a future paper. In the same bed and also in other beds poorly preserved, rather large ostracods were found. Their state of preservation prevents proper identification.

### Invertebrate trace fossil record

Scarce invertebrate trace fossils have been found in the Lower Complex^21,26^. They occur in discrete intervals or levels which are separated by trace fossil-barren intervals. Four ichnoassemblages of invertebrate trace fossils were identified (Fig. 2; see Supplementary Information)^26^. The studied assemblages were found in sparsely distributed horizons and are usually dominated by a single or in some case a few ichnotaxa with locally high trace-densities. The most conspicuous are traces produced by arthropods (probably small crustaceans), which can form horizontal burrows. The invertebrate ichnotaxa recognized until now are well known from non-marine and marginal-marine deposits^26^. Distribution and composition of the invertebrate trace fossil assemblages from the Lower Complex probably reflects occurrence of the impoverished, stressed *Cruziana* ichnofacies^26^. It is worth to mention that the one isolated invertebrate trace fossil assemblage from the track-bearing interval (Z3 with *Rhizocorallium* isp. and cf. *Balanoglossites* isp.) clearly points to deposition in marine-influenced or a marginal-marine, probably lagoonal environment. The Z3 assemblage was only identified in one level, contrasting with other trace fossil assemblages of the Lower Complex, and is interpreted here as the result of an isolated marine incursion (Fig. 2).

Some trace fossils that previously were undescribed from the succession were investigated (see Supplementary Information). One of these was a sinusoidal (i.e. meandering) trace-fossil of variable length. The width of the trace is up to one centimeter. The peak-to-peak amplitude (imaginary vertical line between crest and trough, *i.e*. 2 times the amplitude) range from two to three centimeters and the wave-lengths are between 3–5 centimeters. This ichnofossil resembles that of *Cochlichnus* in appearance but is of a much larger size and slightly irregular.

Another type of structure is composed of faint traces interconnected by nodes and lines of concave epirelief that are composed of small peloid grains. In similarity to paths of modern earth-worms, the tracks are interpreted to have been formed by a worm-like organism, most likely a sediment-feeding annelid. The traces themselves are collapsed but fecal material left behind function as studs that maintain the structure of the trace.

Large burrows (up to 11 cm in diameter) are common elements of the thick dolomicritic beds, and some palaeosol levels, but are also associated with a single layer with tetrapod traces (Fig. 3; see Supplementary Information). All identified burrows were created by animals living at the time of bed formation and are filled with the same dolomitic sediment as the surrounded matrix. They are represented by three forms: 1) horizontal burrows; 2) burrows with two openings and 3) single vertical shafts with a short tunnel and characteristic infill at the top. The most interesting are horizontal, ornamented with deep ridges, short or very elongated burrows which are similar to *Beaconites* traces. These characteristics suggest that the burrows are most likely to be the work of large arthropods such as millipedes or crustaceans^26^.

### Microbially induced sedimentary structures (MISS)

The biotic composition of the Lower Complex is dominated by MISS. These include stromatolites, micro-oncoids and, especially, microbial wrinkle structures of which the *Kinneyia* type is the most common (Figs 2 and 3; see Supplementary Information). Wrinkled surfaces are generally interpreted as irregular surfaces of microbial mats that may have been formed by deformation of the mats or underlying sediment by currents and waves. Such abundancy of microbialites and a low degree of bioturbation implies that a ‘primitive’ cyanobacteria-bacteria-algae complex flourished in the absence of other organisms. Similar scenarios have been shown in the aftermaths of several Phanerozoic extinctions^27–31^ and in harsh environments where extreme or highly variable external factors (e.g., temperature, light, CO_2_ saturation and/or water chemistry), allow tolerant cyanobacteria and other microorganisms to flourish in the absence of grazers. The most common setting for modern microbial mat growth is within a continuum of shallow water environments (e.g., hyper-saline lagoons) through to supra-tidal and even sabkha settings, but numerous examples are known from lakes and rivers as well.

## Discussion

Stable isotope signatures, sedimentological architecture and microfacies suggest that the major part of the Lower Complex^21^ was deposited in short-residence-time closed basins. The major part of the deposit is evidently non-marine, but it is punctuated by two inwash deposits of reworked marine microfossils and what appears to be a brief episode of marine-influenced water conditions. This shows that the sea was not far away, even though the depositional environment was certainly beyond the reach of normal spring tides. Looking, as it were, in the other direction across the landscape, the modest content of terrigenous silt and clay is most easily explained as arriving by aeolian processes^21^ or in water suspension from the closest land area (e.g., Mazury Land). This indicates that the depositional environment of the Lower Complex was located far from high ground, most likely on the seaward edge of a wide, flat coastal plain. It is possible that some small streams entered the depositional basin, but it does not seem to have been connected to any major river system.

Within this landscape developed a succession of short-lived muddy, shallow lakes, one of which apparently became a brackish lagoon as the sea made a brief and limited incursion. The lakes seem to have contained stressed ecosystems of low diversity, dominated by microbial mats, charophytes, and – from time to time – small arthropods. Fish were apparently mostly absent, judging by the absence of fish scales in the sediment, though a single fish swimming trace (ichnogenus *Undichna*^32^; see Supplementary Information) has been recovered from the Lower Complex. Water temperatures appear to have been high, around 30 °C. Cyclic changes to the local climate or water supply periodically caused the lakes to dry out and become replaced by vegetated soils. Data from palaeopedological studies suggest that the vegetation was sparse and composed of moisture-loving herbaceous to small shrubby plants^20^. Large terrestrial or amphibious arthropods, most probably either myriapods or crustaceans, dug their burrows into these soils. It is not clear whether the lakes entirely disappeared during these dry episodes or whether a central, deeper and more permanent, part of the lake basin existed outside the area represented by Zachełmie Quarry. Possible recent analogues of the Lower Complex environment include examples of saline and carbonate lakes that are connected to large lagoons, notably those on Kiritimati Atoll, Christmas Islands, central Pacific. However, there are important differences between the Lower Complex and these modern analogues. For example, microbial mats are not as prominent in recent lakes, and the precipitated carbonate minerals in the Lower Complex appear to have been dolomitic rather than calcitic or aragonitic as in the recent examples.

Just above the track-bearing interval, a transition from clayey dolomitic mudstones to abundant wackestones and clayey dolomicrites, accompanied by the disappearance of desiccation indicators and the first appearance of marine body fossils, marks the transition into the Upper Complex. This transition represents the incursion of marine waters, most likely as an initial lagoonal phase which terminated in the development of amphiporoid-dominated lagoons in the lower part of the Kowala Formation.

What were tetrapods doing in the Lower Complex environment, and what does their presence there tell us about their capabilities? All known elpistostegids and Devonian tetrapods are predators with somewhat crocodile-like heads and dentitions suitable for capturing relatively large prey, so it is probably safe to assume that the Zachełmie trackmaker conformed to this general morphotype. The preserved tracks and trackways were made underwater, but it seems rather unlikely that an impoverished aquatic ecosystem like the Lower Complex lakes could have sustained predators reaching two metres or more in length. More probably the tetrapods sought their food elsewhere and made intermittent use of the lakes for other purposes, such as rehydration or breeding; the latter is an intriguing possibility in view of the apparent lack of predators in the lake environment. The record of a brief marine-influenced, probably lagoonal phase (ichnoassemblage Z3) in the Lower Complex suggests a tolerance of marine conditions in the tetrapods, a characteristic that has also been inferred for Late Devonian tetrapods based on their wide distribution across Laurussia and Gondwana and their occurrence in tidally influenced deposits^33^.

The combined characteristics of the Lower Complex lake environment and its setting in the landscape suggest that, even though the tetrapod tracks are subaquatic, the tetrapods themselves must have been capable of terrestrial locomotion. Possible food sources in the area include the burrow-making terrestrial arthropods, a range of fish and invertebrate prey in the adjacent shallow marine environment, and dead or moribund prey that could be scavenged in the intertidal zone; but none of these could be reached from the Lower Complex lakes without at least a short overland journey, and some of course involve prey capture on land.

Recent decades have seen a lively debate about the degree of correlation between terrestrialization and the evolution of tetrapod morphology. Evidence from the Late Devonian genus *Acanthostega* has been used to argue that tetrapods are primitively aquatic^34^, but others have pointed out that the anatomically more primitive humeri of *Ichthyostega* and the un-named Devonian tetrapod ANSP 21350 from Pennsylvania show obvious weight-bearing adaptations, implying terrestrial capability in potentially less crownward animals^35,36^. The palaeoenvironmental, palaeoecological and palaeoichnological data from the Lower Complex of the Wojciechowice Formation offer circumstantial support for the early terrestrialization of tetrapods. The tracks were made by paired appendages with digits and discrete sole pads^1^, very different from the fins of lobe-finned fishes; the environmental and ecological setting suggests that the trackmaker used these appendages on land as well as in water. Thus, dissimilar to the intertidal, woodland or lagoonal scenarios the proposed ephemeral lakes model of a quadrupedality development draws upon the utility of limbs not only within shallow water environments but also during locomotion outside water habitats.

Trackway and footprint data, for all their limitations, cast unique and direct light on the lives of the trackmakers. In forthcoming papers we will examine the information that other Devonian tetrapod track sites provide about the terrestrialization and diversification of the group.

## Material and Methods

### Microfacies analysis

In total, thirty-four thin sections were made from rock samples collected from the track-bearing horizons as well as overlying and underlying beds. These were produced by mounting slabs onto glass slides using epoxy, and subsequently grinding them to a suitable thickness. The thin sections were studied using a binocular microscope coupled with a digital camera as well as with an optical microscope (NIKON Eclipse LV 200).

### Scanning Electron Microscope and Energy-Dispersive X-ray Spectroscopy

Two samples from each of the three track-bearing horizons were prepared for analyzes with a Zeiss Supra35VP FEG Scanning Electron Microscope (SEM) equipped with an EDAX Apollo X Energy-Dispersive X-ray Spectroscopy detector (EDS). Preparation consisted of breaking around 0.5 cm^2^ big pieces of fresh rock that were glued onto pin stubs. These were subsequently coated with a gold/palladium (Au/Pb) alloy in order to enhance imaging. The coating was accounted for in the EDS analyzes by the subtraction of Au/Pb and any other associated peaks in the spectra that the coating were blamable of.

### Specimens and photo documentation

Plates were made from photographs of collected material and the field site. Images were processed in Photoshop using contrast/brightness, noise reduction and minor color level changes. The background in some pictures of slabs were removed and replaced by a black color in order to make clear illustrations.

### Bed identification

The nomenclature for bed identification follows that of Narkiewicz *et al*.^21^ as far as possible. However, since some beds are missing and several beds are included under the same bed, another system is also inferred across the track-bearing horizons. Names of this system are composed by two letters indicating the track-bearing interval in question (e.g., TB = track-bearing Horizon B) followed by a letter indicating above or below (U/L) the horizon and a number indicating how many beds away from the track-bearing horizon; 0 being the immediate neighboring bed. As an example, a bed named TC U1, thus refers to the bed two beds above track-bearing Horizon C.

### Rare earth elements (REEs)

The concentrations of REEs were normalized to the average Post-Archean Australian Shales (PAAS) of Taylor and McLennan^37^ in order to remove abundance effect and show eventual deviations from the average shale composition. Furthermore, the determination of Ce and La anomalies follows the scheme of Bau and Dulski^38^. This technique is made by comparing (Ce/Ce*)SN = [2Ce/(La + Pr)]SN to (Pr/Pr*) SN = [2Pr/(Ce + Nd)]SN in order to avoid calculating false cerium anomalies, that may actually be based on the presence of La anomalies. By doing so, one counts for an absence of Nd and Pr anomalies.

### Data Availability

All data generated or analysed during this study are included in this published article (and its Supplementary Information files).

## Electronic supplementary material

supplementary material
