# Supplementary material for: Non-marine palaeoenvironment associated to the earliest tetrapod tracks
Source: Sci Rep. 2018 Jan 18;8:1074. doi: 10.1038/s41598-018-19220-5 (PMC5773519; doi:10.1038/s41598-018-19220-5)
Supplement: Supplementary file 1 — supplementary material [file 41598_2018_19220_MOESM1_ESM.pdf]

SUPPLEMENTARY INFORMATION

Non-marine palaeoenvironment associated to the earliest tetrapod tracks

Martin Qvarnström[1], Piotr Szrek[2], Per E. Ahlberg[1] and Grzegorz Niedźwiedzki[1*]

[1]Subdepartment of Evolution and Development, Department of Organismal Biology, Evolutionary Biology Centre, Uppsala University, Norbyvägen 18A, 752 36 Uppsala, Sweden
[2]Polish Geological Institute-National Research Institute, Rakowiecka 4 Street, 00-075 Warszawa, Poland

*Corresponding author - grzegorz.niedzwiedzki@ebc.uu.se

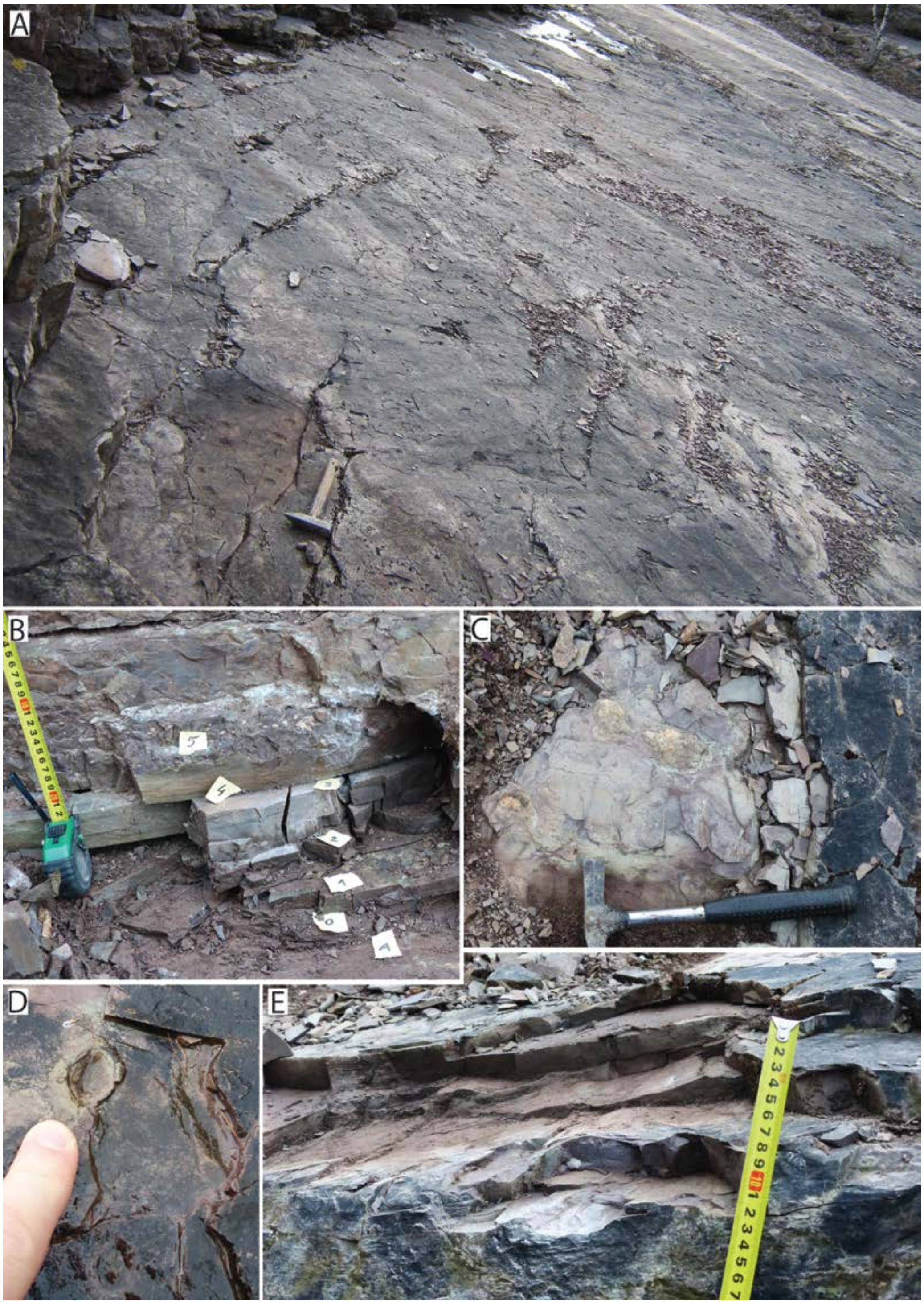

Figure 1. Field photographs of Horizon A. **A)** Overview of the horizon with a trackway just above the hammer which is around 30 cm long. This particular trackway has since been removed from the quarry and is stored at the Geological Institute of Warsaw (Muz. PGI 1728.II.16). Note the irregular surface of the horizon and the presence of widely spaced wave ripples. **B)** Horizon A in the lowermost part of the picture, with overlying beds. Note the thin clayey bed just above the track-bearing horizon and the potential palaeosol (topmost numbered bed) with large white nodules and erosional base. **C)** Stromatolites found just below Horizon A. **D)** Enigmatic structure just below Horizon A (on the same level as the stromatolites). **E)** The beds underlying Horizon A (topmost bed on picture). Note the evident ripple marks in the lower beds.

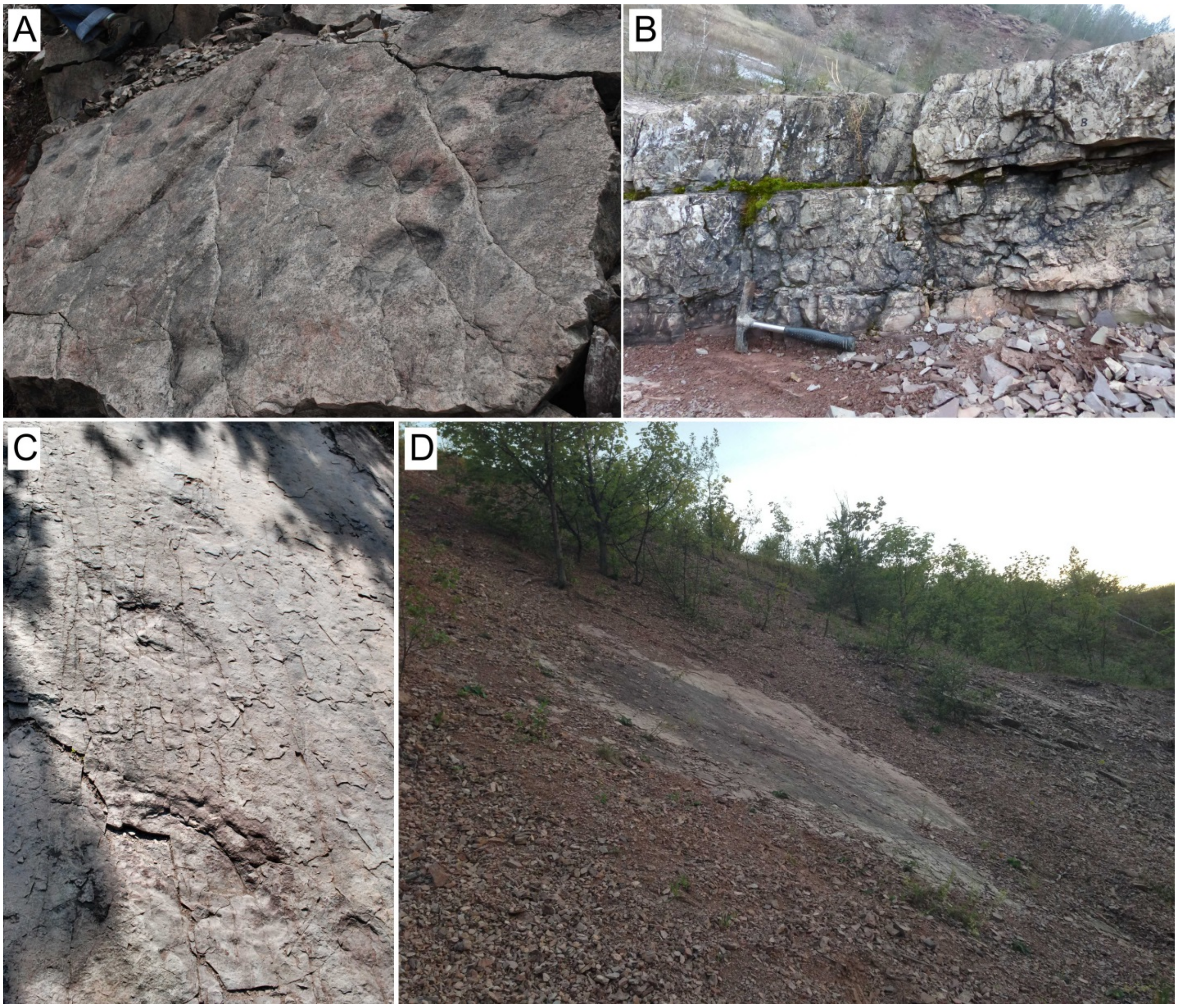

Figure 2. Field photographs of Horizons B and C. **A)** Horizon B with tetrapod tracks and track-ways. **B)** The beds underlying Horizon B (topmost surface). Note the very high clay content of the lowermost bed on which the hammer rests. **C)** Large tracks with digit impressions preserved on top of Horizon C. **D)** Overview of Horizon C in the western of the Zachełmie Quarry. The overlying beds are visible in the middle right part of the picture.

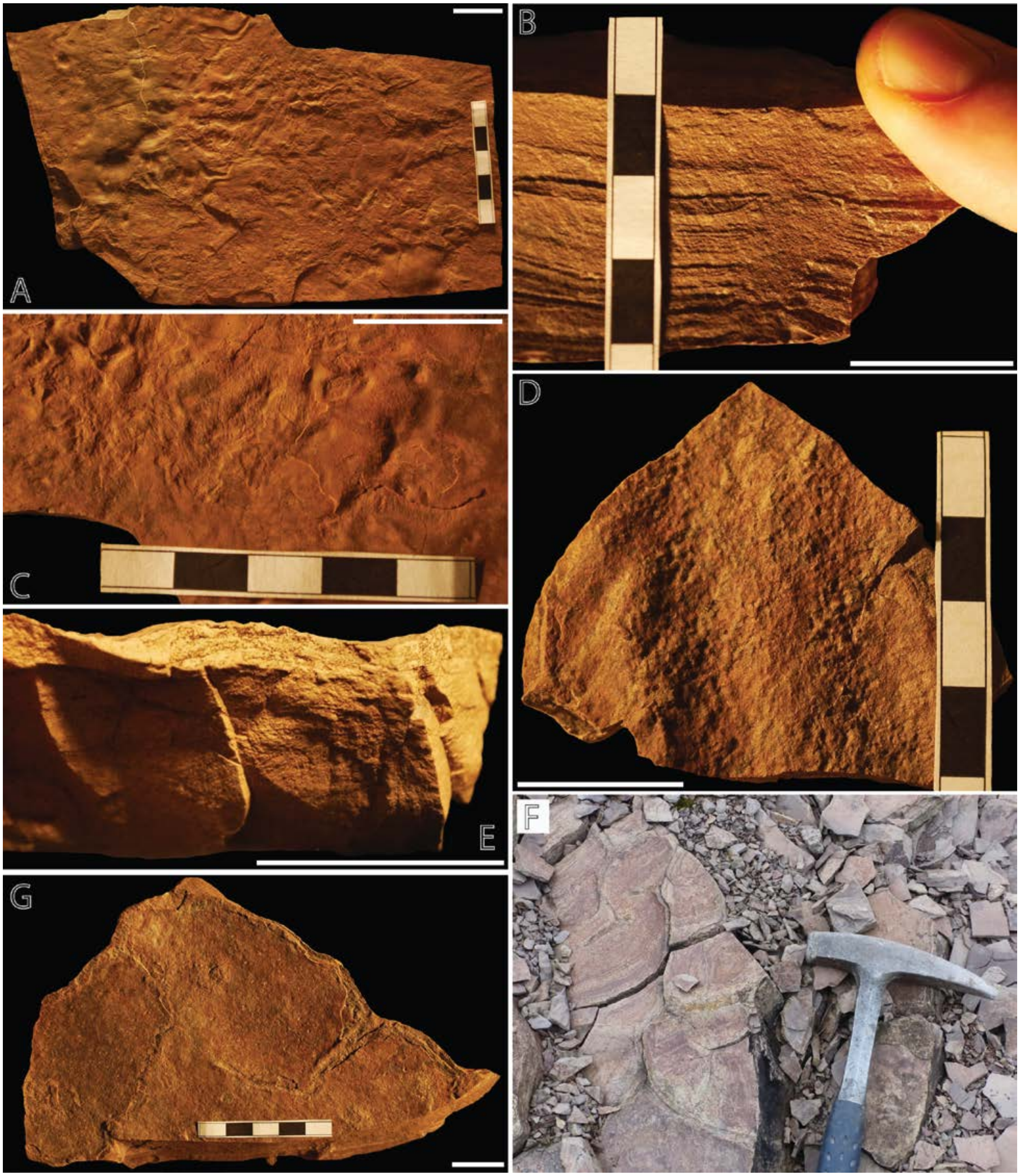

Figure 3. Photographs of sedimentary features of the Lower Complex of the Wojciechowice Formation. **A)** and **C)** Deformed microbial mat from bed 26-27 EM in Narkiewicz et al.[21]. In the magnified picture (C) it is possible to see the characteristic wrinkles preserved in the mat. **B)** Cross-section of the boundary between two desiccation polygons. The crack is resealed but shows a typical V-shaped structure. **D-E)** Wave ripple marks from above and in cross-section. **G-F)** Photograph of slab and field photograph of subaerial surface with desiccation cracks. If not indicated, the samples are collected as free-lying slabs (*ex situ)* from the Lower Complex.

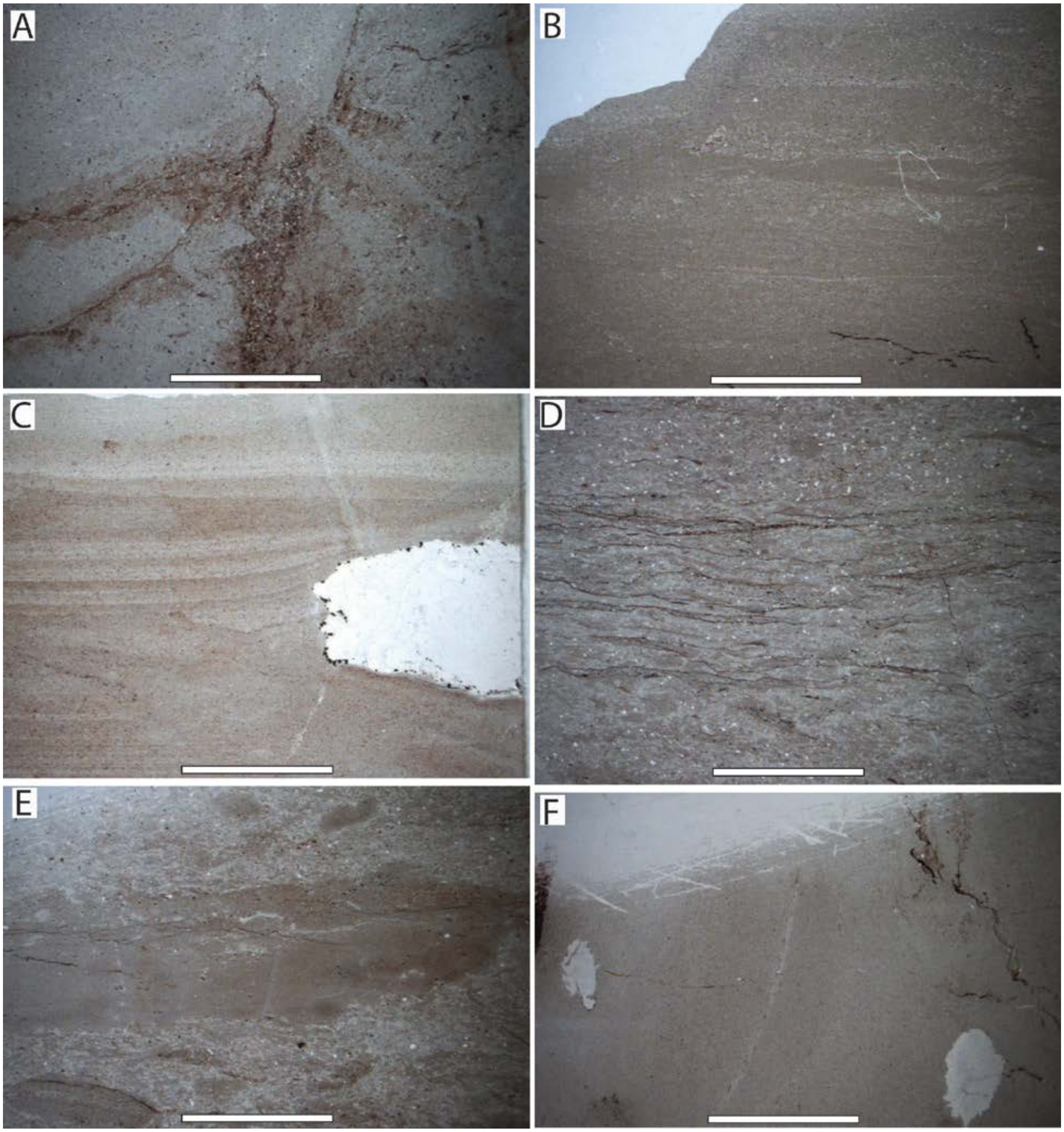

Figure 4. Images of thin sections (from binocular microscope) displaying different microfacies. **A)** Breccia/Conglomerate microfacies with possible root trace (bed: TB L2). **B)** Planar finely laminated clayey dolomitic shale. Note micro-erosion or sediment bioturbation in the topmost part and the clayey layer with detrital lenses, similar to a fluvial deposit. (Horizon C**). C)** Abiotic laminite affected by synsedimentary growth of supposed evaporite minerals that now occur as pseudomorphs (Horizon A). **D)** A relative 'high'-energy deposit with sand-sized quartz grains, possible micro-oncoids and abundant seams from pressure-dissolution of carbonate minerals (bed: TB U4). **E)** Possible micro-oncoids forming an oncoidal wackestone. This microfacies was formed in the highest depositional energy (compared to all samples) and is interpreted to have been formed in the lake margin (bed: TB U3). **F)** Carbonate nodules in a homogenized matrix from the proposed paleosol level (bed: TA U5**).** Scale bars equal 5 mm.

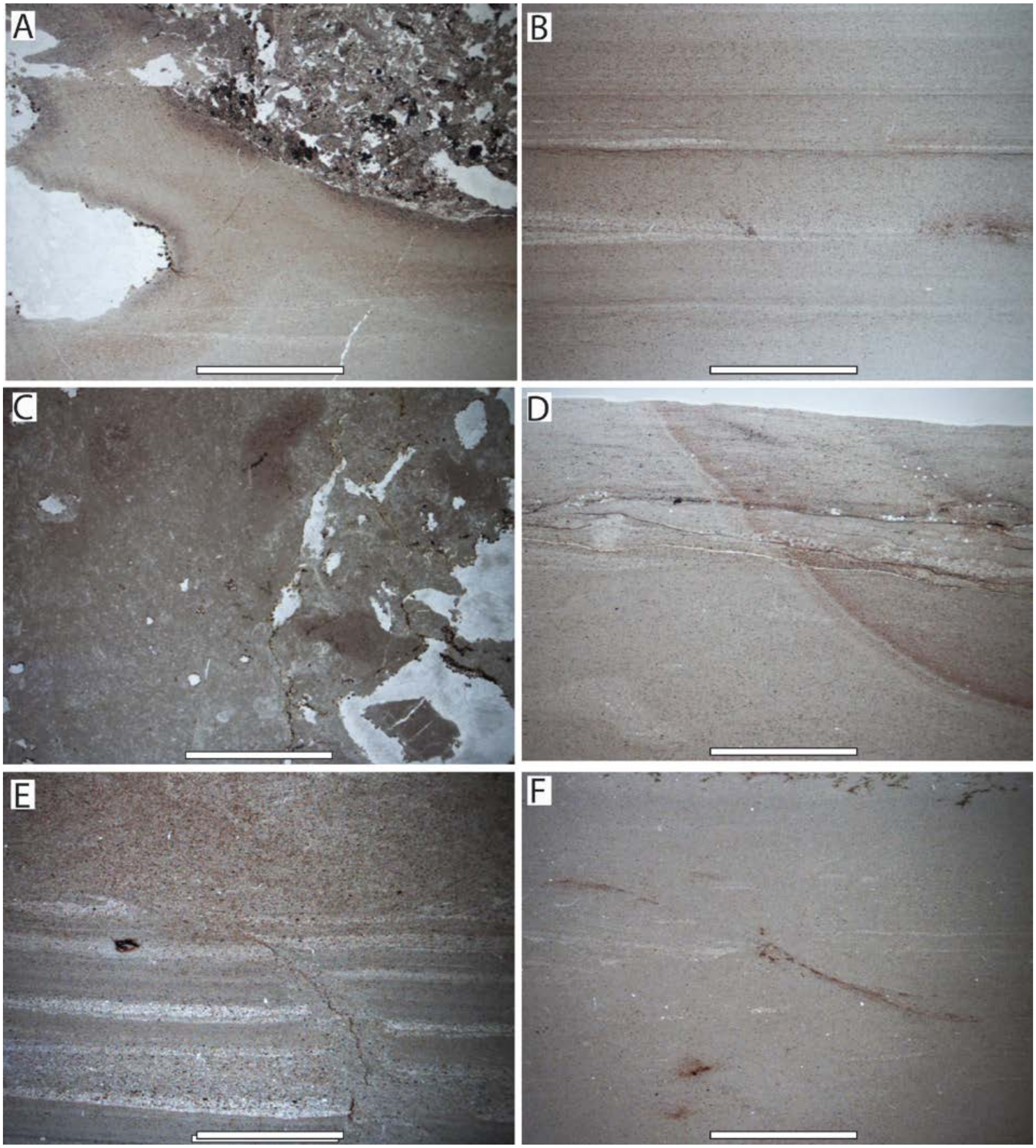

Figure 5. Images of thin sections (from binocular microscope) displaying different microfacies. **A)** Lowermost part of stromatolite. Note the internal fenestral structures, large void in sediment in the left part of the image and sediment compaction under the stromatolite loading on the sediment (bed: TA L0). **B)** Fine planar lamination which characterizes the microfacies abiotic laminite. **C)** Oncoidal wackestone **(**Horizon B). Note recrystallized void, which is a part of a larger structure interpreted as a microoncoid in the topmost left part of the picture. Cracks and mineralized fenestral structures are seen in the bottommost right part. **D)** The lower part of the section is bioturbated, reworking resulting in the absence of primary lamination. Stylolites in the tomost part show accumulation of terrigenous clays and larger sand-sized quartz-grains. Note the micro-sized domal stromatolite in association to the lower stylolites (bed: TA L2). **E)** Abiotic lamination with light detrital and dark more peloid-rich laminae. Note oblique burrow or root trace that penetrates the laminae and the reworked upper part of the section (bed: TA U2). **F)** Mudstone with rare inclusion of light detrital matter (bed: TA U2). Scale bar equals 5 mm.

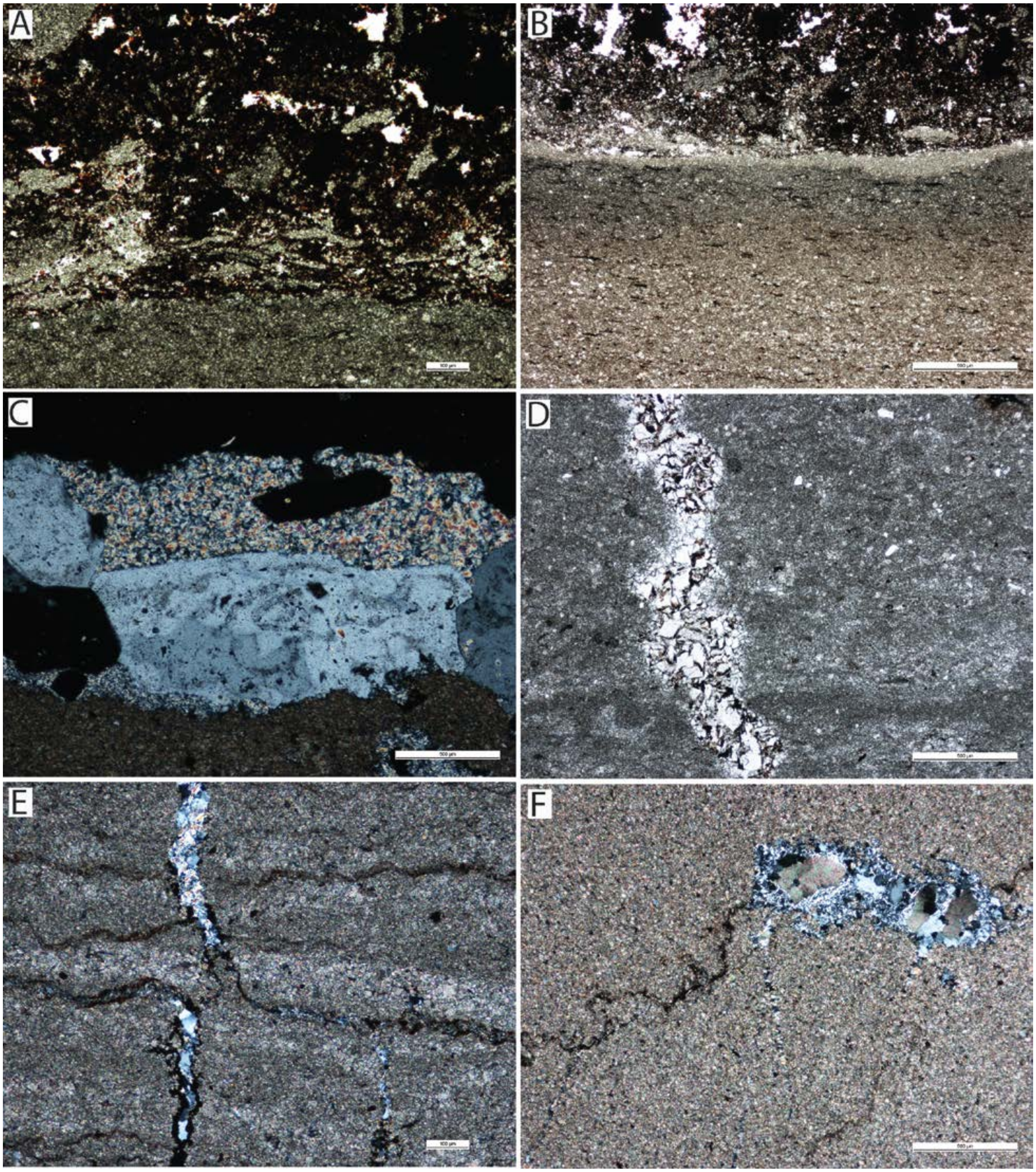

Figure 6. Images of thin sections (from optical microscope). **A)** Lowermost part of stromatolite (bed: TA L0) and underlying sediment. Note the cyanobacterial filaments that are preserved in the middle part of the picture. **B)** Sediment reaction under the stromatolite, perhaps due to early compaction caused by overlying structure (bed: TA L0). **C)** Quartz, hematite in association with anhydrite and gypsum in a filled 'void' from Horizon A. **D)** Crack filled with carbonate minerals and associated hematite in an oncoidal wackestone (bed: TB U0). **E)** Cracks and stylolites in a microbial laminite (bed: TB U1). **F)** Stylolite and quartz/carbonate precipitation in void (bed: TB UO). Scale bars equal 100 (A & E) respectively 500 (B, C, D & F) µm.

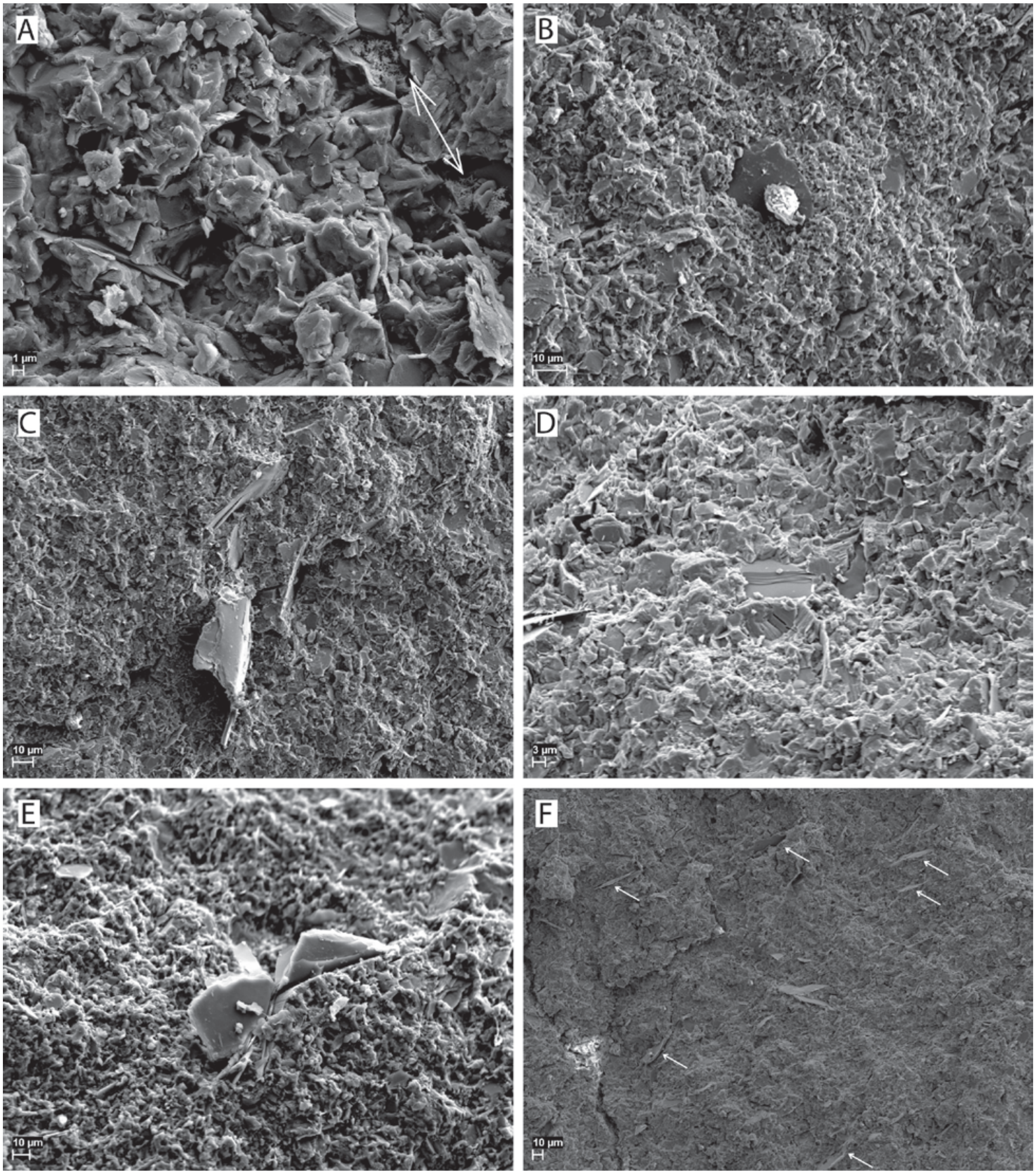

Figure 7. Scanning electron microscope photographs of sediments from the track-bearing horizons. The pictures derive from: Horizon A (pictures A-C); Horizon B (picture D), and; Horizon C (pictures E-F). **A)** This fine-grained, homogeneous dolomitic matrix is characteristic for all three horizons. Arrows indicate rosette-shaped minerals that are interpreted as pore-filling clays. **B)** A siliciclastic grain embedded in the dolomitic matrix. At the surface of the large grain there is an enigmatic colonization of small white minerals. **C)** Possible shell fragments in fine dolomitic matrix. **D)** Embedded siliciclastic grain with numerous enigmatic dark ribbons. **E)** Potassium aluminosilicate grains embedded in a fine-grained matrix. **F)** A clustering of silt-sized siliciclastic grains. Arrows indicate a preferred orientation of the elongated grains which likely represent higher energy conditions (i.e. currents or waves) at the time of deposition.

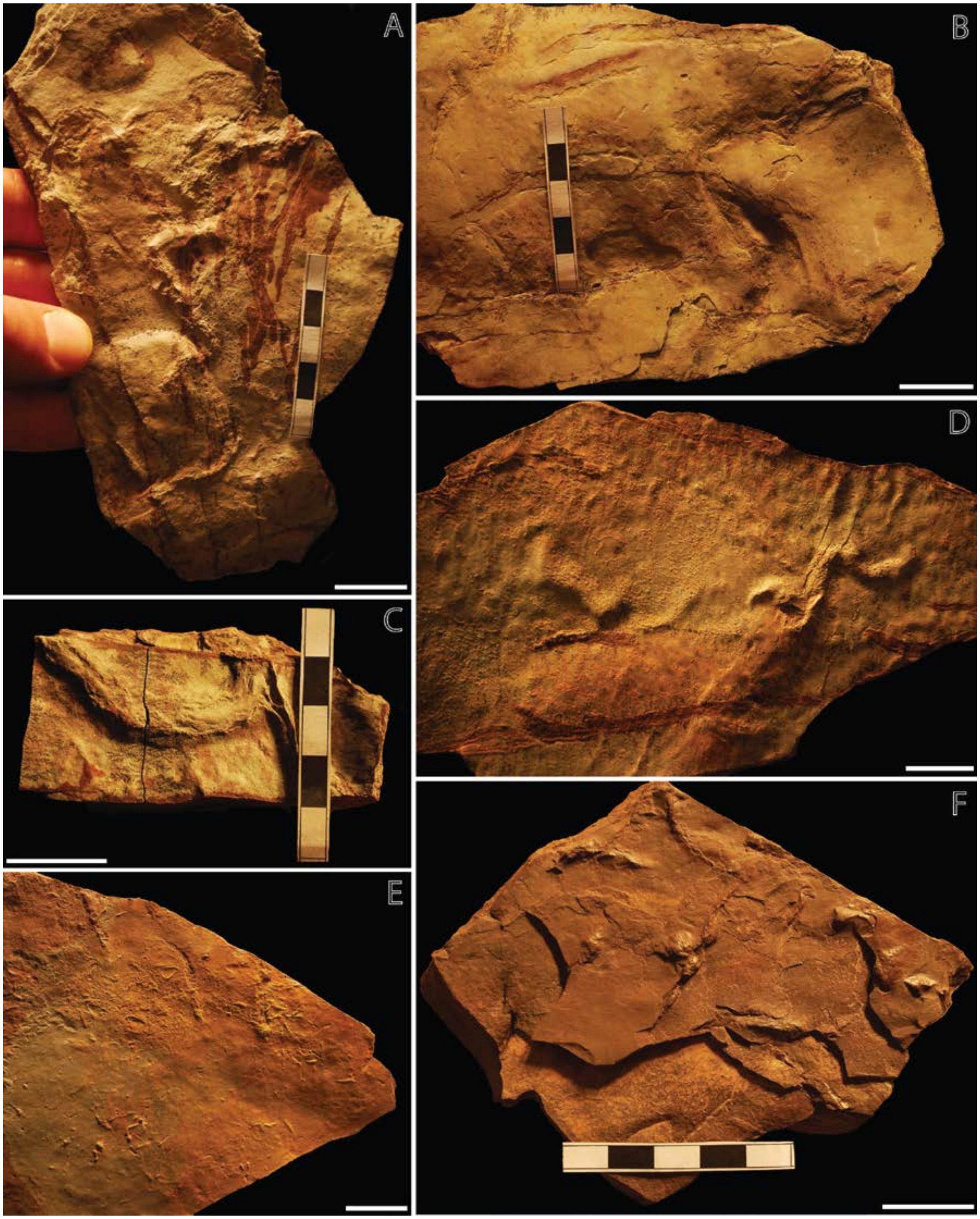

Figure 8. Previously undescribed invertebrate ichnofossils from the Lower Complex of the Wojciechowice Formation. **A-D)** Sinusoidal or slightly irregular meandering trace-fossils of about 0.5 to 1 centimeter in thickness. The wavelength varies from 3-5 cm and the width from 2-3 cm. The ichnofossils might represent large *Cochlichnus*. **E)** Small burrows interpreted as having been formed by small invertebrates (e.g., nematodes). Note bigger trace fossils in topmost part of the image, which cut and terminate the small burrows. Some shells belonging to bivalves or large ostracodes are seen in the upper part of the picture (slab derives from bed 26 EL from Narkiewicz et al.[21]). **F)** Invertebrate trace fossils in dolomitic shales. The tracks are composed of peloid-filled nodes that are interconnected with poorly preserved burrows. The peloids might represent the fecal matter of a large burrowing annelid. Similar peloidal invertebrate trace fossils are also found in bed 10 EL. All other slabs were collected as free-lying slabs belonging to the Lower Complex. Scale bars equal 2 cm.

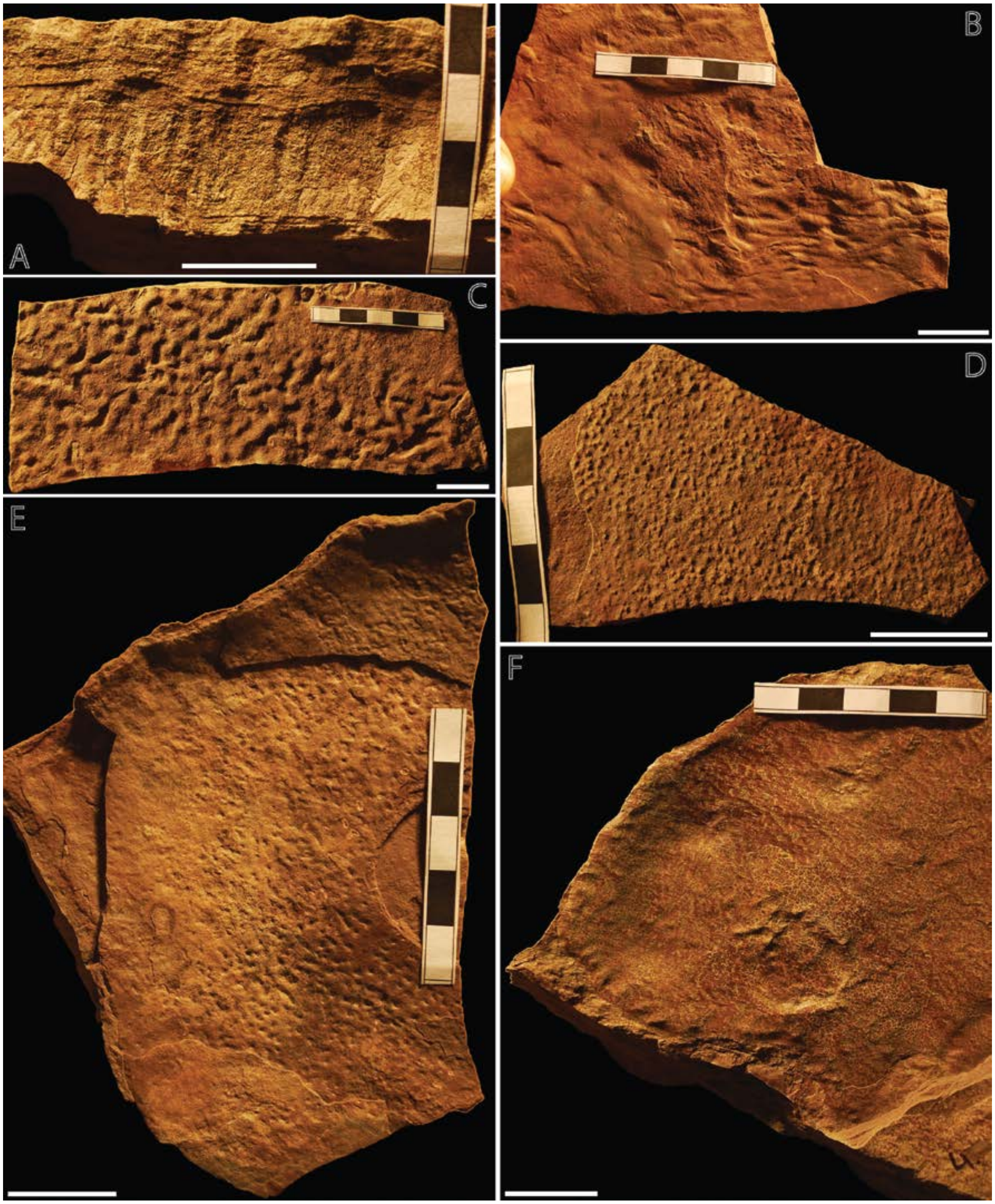

Figure. 9. Microbially-induced sedimentary structures (MISS). Cross-section (**A**) and surface (**C**) of a slab with deformed microbial wrinkle structure (bed no. 26 EL). **B**) Microbial mat, in part showing microbial deformation structures (lower right). **D**) Microbial structures, perhaps representing gas escaping hollows. **E**) Mud cracked polygon covered with a bacterial mat with small holes (bed no. 35 EM). These might also represent gas escape structures, perhaps made from oxygen produced by the mat itself. **F**) Slab with microbial mat covering an enigmatic structure, collected from bed 35 EM. The border zone between the microbial mat and surrounding sediment is clearly visible, reflecting the growth rim of the mat. All samples except (**F**) were collected as free-lying slabs belonging to the Lower Complex. Similar *Kinneyia* type structures as in (**C**) are found in bed 26 EL and analogous deformed microbial mats as in (**B**) in 26-27 EM. Scale bars equal 2 cm.

SUPPLEMENTARY INFORMATION

Non-marine palaeoenvironment associated to the earliest tetrapod tracks

Martin Qvarnström[1], Piotr Szrek[2], Per E. Ahlberg[1] and Grzegorz Niedźwiedzki[1*]

[1]Subdepartment of Evolution and Development, Department of Organismal Biology, Evolutionary Biology Centre, Uppsala University, Norbyvägen 18A, 752 36 Uppsala, Sweden
[2]Polish Geological Institute-National Research Institute, Rakowiecka 4 Street, 00-075 Warszawa, Poland

*Corresponding author - grzegorz.niedzwiedzki@ebc.uu.se

Tables 1-3. The tables contain microfacies description of each horizon as well as neighboring beds. Lamination is noted together with an interpretation of depositional energy and palaeoenvironmental significance. The ID represents the level in relation to the track/bearing horizon (i.e. U0 being the horizon directly above). Abbreviations; Qtz- quartz, Hem- Hematite, Do-Dolomite. Note that the depositional energy indications are very relative since all beds are characterized by fairly low depositional energies (high clay content and small grain sizes); however, it is useful to distinguish the conditions that applied for the individual beds to understand the temporal development.

Table 1. Track-bearing horizon A.

ID (horizon A)	Microfacies	Lamination/Fabric	Depositional Energy	Palaeoenvironmental Interpretation	Additional comments
**U5**	Palaeosol	None. Homogeneous and massive.	Subaerial (?) reworking	Subaerial palaeosol	The sample exhibits numerous 'voids' or inclusions with recrystallized mineral phases (Qtz, Do, Hem) having both sharp and irregular boundaries (*i.e.* both appear in 'equilibrium' and seem 'degraded'). Numerous sediment-infilled cracks (with Hem accumulation) may very well represent root traces. Black or dark "grains" and small patches of carbonate occur in otherwise fine-grained, reworked and homogenous sediments with some scarce siliciclastic grains.
**U4**	Abiotic laminite	Planar	Low	Shallow lake floor rarely affected by waves	Mostly undisturbed planar lamination of different thickness with lighter detrital laminae often displaying erosional lower boundaries. Darker laminae are composed of peloids and some scarce siliciclastic grain even though much at much lower rates than in light laminae.
**U3**	Abiotic laminite	Planar to wavy fine-laminated	Low	Lake floor rarely affected by waves	Lamination is disturbed by microerosional boundaries. 'Voids' with Do, Qtz and Hem present.
**U2**	Abiotic laminite	Planar to disturbed by reworking	Low	Lake floor rarely affected by waves	Parts of the thin section display reworking of the sediment probably linked to bioturbation or plant activity. Reworked parts are massive in contrast to the abiotic lamination which in part is preserved.
**U1**	Abiotic laminite	Planar to disturbed by reworking	Low	Lake floor rarely affected by waves	The upper part of the thin section display reworking of the sediment probably linked to bioturbation (less bioturbation than in U2). Reworked parts are massive in contrast to the abiotic lamination which in part is preserved. Some 'voids' are present and contain Do and idiomorphic Hem.
**U0**	Mudstone (laminated)	Planar fine-laminated, some cross-bedding	Very low	Lake floor rarely affected by waves. Stagnant conditions.	A very clay-rich bed with dispersed, small lenses of lighter detrital laminae.
**A**	Abiotic laminite	Planar fine-laminated	Low	Lake floor rarely affected by waves	Microbial laminate with some sparry fossil fragments. 'Voids' with Qtz, Hem (and anhydrite and gypsum?).
**L0**	Abiotic laminate	Planar fine-laminated. Lenses of detrital material. Topmost part (stromatolitic) bindstone	Low	Lake floor rarely affected by waves	Very fine lamination. 'Voids' with irregular boundaries occur with interiors filled with recrystallized Do, Qtz and, especially, Hem. An enigmatic structure with Qtz recrystallization represents a small burrow. Some accumulations of fragmented and indeterminable fossil fragments which might represent wash-up 'events'. Stromatolitic structures (topmost part of the bed); supposed cyanobacterial filaments visible in the base of the structures which are otherwise extremely recrystallized with both euhedral and 'degraded' crystals, resembling those of other 'voids' but with more iron oxides. Iron oxides are everywhere, indicative of organic material. The non-remineralized parts are blocks with preserved microbial spheres. The blocks are oriented in different directions, but internal structure maintained.
**L1**	Abiotic laminate	Planar	Low/moderate	Lake floor partly affected by waves	Laminae often pinching out and cut by erosional truncations. Small accumulations of siliciclastic sand grains witness of some current, wind or wave energy increase (probably the latter). Some enigmatic structures are found that perhaps are the result of bioturbation. Small stylolites or seams are in part fairly well-developed.
**L2**	Abiotic laminate	Massive with lenses of light laminae and faint dross bedding	moderate	Lake floor partly affected by waves	Seams or stylolites occur with associated accumulation of Qtz grains of fine to medium sand size. These are subangular to subrounded. Lenses of lighter detrital sediment occur as well as faint micro cross bedding.
**L3**	Abiotic laminate	Planar to crinkled fine-laminated	Low	Lake floor partly affected by waves	Alternating light and dark laminae, the former often with grains fining upward resting on a microerosional base. Big void on slab.

Table 2. Track-bearing horizon B.

ID (horizon B)	Microfacies	Lamination/ Fabric	Depositional Energy	Palaeoenvironmental Interpretation	Additional comments
**U4**	(?Micro-oncoidal) Wackestone	Chaotic	Relatively high	Wave-affected lake margin	Siliciclastic grains including abundant Qtz grains up to the fine-grained sand fraction and some micas (muscovite) are dispersed evenly through the sample. The Qtz grains are sub-angular to sub-rounded indicating a relatively short transport and a 1st order sediment recycling. Seams, marked by accumulations of insolubles are abundant but not very well-developed. Blackened clasts occur as well as round structures composed of fine-grained dolomite representing, perhaps, recrystallized indeterminable fossils.
**U3**	(?Micro-oncoidal) Wackestone/Pack stone	Chaotic	Relatively High	Wave-affected lake margin	Centimeter-sized ‘clasts’ containing micrite are, in part of the section, closely packed. Qtz grains and other siliciclastics up to the fine-grained fraction are dispersed evenly. Massive layers might represent microbial sediment bounding.
**U2**	Microbial laminate	Planar to wavy fine-laminated to chaotic.	Low/moderate	Lake floor rarely affected by waves	Initial lamination is disturbed by bioturbation, tepee structures (?) and development of microbial communities.
**U1**	Abiotic laminite	Planar fine-laminated. Partly disturbed	Low	Lake floor rarely affected by waves	Lamination is very fine, disrupted in the lower part by structures similar to small tepee structures. Lighter laminae consist of larger detrital grains (mostly Qtz) that may reach up to the fine-sand fraction. Sets of seams (not fully developed stylolites) are found parallel to bedding. Cracks (some possibly representing root traces) occur perpendicular, parallel and oblique to bedding and are sediment infilled, marked by hematite and/or contain precipitated carbonate. Clusters of hematite after possible organic structures are dispersed in the sediment.
**U0**	Wackestone	Chaotic	Low + reworked?	Lake margin with reworking of material	A crack runs through the whole sample with an interior dolomite precipitation in association to some hematite. The bedding is reworked resulting in a chaotic non-laminated appearance of the sediment. In the topmost part of the thin section there is a set of seams associated with more abundant and bigger siliciclastic grains (Qtz, Musco, Do). Local accumulations of iron oxides might be after organic matter. Round blackened small objects. Cloudy appearance in general due to high clay content. Root traces (?).
**B**	(?Micro-oncoidal) Wackestone	Chaotic	Low + reworked?	Lake margin with reworking of material	Very abundant small and big ‘voids’ filled with secondary crystallization of Do (major) and Qtz as well as a nearly opaque phase (Hem). Small anhydrite grains in association to dolomite in voids. The sediment has a cloudy-looking appearance in general, similar to L0 but also containing blackened clasts and other round grains with micrite replaced interiors.
**L0**	Wackestone, ?Palaeosol	Chaotic	Low	Lake margin with reworking of material	Cracks occur which are either sealed (+Hem accumulation), sediment infilled or contain recrystallized Qtz/D. Some may represent root traces. The organization is chaotic (*i.e.* very disturbed) but not homogenous. Reworked white ‘clasts’ represent the former detrital laminae. Cloudy appearance due to high clay content.
**L1**	Wackestone	Planar to wavy fine laminated	Low, reworked	Lake floor rarely affected by waves	Faint lamination with brighter layers of detrital accumulations. Sets of seams parallel to bedding indicate of post depositional pressure-dissolution.
**L2**	Grainstone ?Palaeosol	Massive	Low/Subaerial (?) reworking	Possible subaerial paleosol	Larger ‘grains’ with micritic interior appear in this bed. They are found in association to cracks/root traces infilled with siliciclastic grains (mostly Qtz).
**L3**	Abiotic laminite	Planar fine-laminated (disturbed)	Low/moderate	Lake floor partly affected by waves	Similar in appearance to L4 but with more irregular laminae. Light laminae are, in part, crinkled and irregular.
**L4**	Abiotic laminite	Planar fine-laminated (disturbed)	Low	Lake floor rarely affected by waves	Light laminae of different thickness of which thicker have erosional base toward darker laminae. Sealed cracks representing root traces?

Table 3. Track-bearing horizon C.

ID (horizon C)	Microfacies	Lamination/Fabric	Depositional Energy	Palaeoenvironmental Interpretation	Additional comments
**U2**	Mudstone	Scarce lenses of detrital matter	Very low	Lake floor rarely affected by waves	The light detrital matter is mostly confined to small lenses that are sparsely distributed. Some horizons, however, are continuous and represent small episodes of deposition of coarser material. Some fine-sand to silt grains of Qtz are also rare but widespread in the otherwise muddy sediment.
**U1**	Mudstone	Scarce lenses of detrital matter and some detrital laminae	Very low	Lake floor rarely affected by waves	Like above with, perhaps, a slight bigger input of detrital grains resulting in a faint lamination.
**U0**	Mudstone	Planar	Very low	Lake floor rarely affected by waves	There are a couple of oblique cracks in the thin section with Qtz and Do recrystallization and He coating. Some structures which might be the result of biogenic activity are infilled with slightly coarser material.
**C**	Mudstone/abiotic laminate	Faintly cross-bedded/planar to wavy lamination	Very low	Lake floor rarely affected by waves	Horizon C is composed of very clay-rich sediment with some inclusions of the light detrital material, mostly located in lenses but also in some distinct laminae.
**L0**	Abiotic laminite	Disturbed planar lamination	Low	Lake floor rarely affected by waves	Faint lamination and seams (perpendicular and parallel to bedding) characterize the sample. An enigmatic thin structure penetrates the whole thin section and differs by not having any iron oxides. Its irregular pattern suggests that it is either a function of bioturbation or a fluid reaction boundary from diagenesis. At least it was made prior to crack formation since a crack (with secondary precipitation of Qtz) cuts through the structure and acts like a normal fault.
**L1**	Abiotic laminite/wackestone	In part deformed fine-laminated	Low to medium	Lake floor rarely affected by waves	Alternating light detrital and dark peloidal laminae. Lamination Is not horizontal but disturbed from loading. It grades into a wackestone which lack lamination.

Table 4. REEs and other trace element concentrations in the petrographical samples (29/15/1-10) from the track-bearing horizons, the Upper Complex of the Wojciechowice Formation, and the Kowala Formation.

No. of sample	Sc	Y	La	Ce	Pr	Nd	Eu	Sm	Gd	Tb	Dy	Ho	Er	Tm	Yb	Lu	Th
	mg/kg																
29/15/1	5,9	9,6	11,0	24,7	2,9	11,3	0,50	2,33	2,00	0,32	1,89	0,37	1,07	0,14	0,93	0,13	4,01
29/15/1	6,0	9,5	11,6	25,7	3,1	11,8	0,50	2,46	2,23	0,34	1,91	0,37	1,06	0,14	0,97	0,14	4,09
29/15/1	6,2	9,7	11,0	25,8	3,0	11,8	0,52	2,38	2,19	0,33	1,92	0,37	1,06	0,15	0,91	0,14	4,03
29/15/1	6,7	10,0	11,4	25,4	3,0	11,8	0,52	2,38	2,15	0,33	1,88	0,38	1,11	0,14	0,93	0,14	4,00
29/15/1 prim	6,0	9,5	11,3	25,6	3,1	11,8	0,48	2,29	2,03	0,31	1,83	0,36	1,03	0,15	0,92	0,13	4,07
29/15/1 prim	6,0	9,6	10,7	25,1	3,0	11,8	0,54	2,37	2,18	0,34	1,88	0,37	1,07	0,14	0,98	0,14	3,92
29/15/1 prim	6,2	10,0	11,4	25,4	3,0	11,8	0,51	2,38	2,15	0,33	1,88	0,37	1,11	0,14	0,95	0,14	4,00
29/15/1 prim	6,7	9,9	11,0	24,8	3,0	11,7	0,52	2,43	2,29	0,33	1,94	0,38	1,11	0,14	0,93	0,14	3,95
29/15/2	4,5	6,6	8,0	17,1	2,0	7,5	0,30	1,45	1,26	0,20	1,20	0,25	0,75	0,10	0,66	0,10	3,04
29/15/2	4,3	6,4	8,3	17,5	2,1	7,9	0,31	1,52	1,39	0,21	1,23	0,24	0,76	0,11	0,65	0,10	3,05
29/15/2	4,8	6,7	8,2	18,4	2,2	8,2	0,32	1,54	1,38	0,21	1,31	0,26	0,78	0,10	0,67	0,11	3,07
29/15/2	5,4	7,0	8,6	18,0	2,2	8,1	0,34	1,55	1,46	0,22	1,33	0,27	0,84	0,10	0,73	0,10	3,11
29/15/2 prim	4,5	6,6	6,8	15,3	1,9	7,5	0,31	1,49	1,32	0,20	1,22	0,24	0,76	0,10	0,65	0,10	3,05
29/15/2 prim	4,5	6,5	7,3	16,2	2,0	7,9	0,34	1,59	1,47	0,21	1,34	0,26	0,79	0,10	0,69	0,10	3,14
29/15/2 prim	4,8	6,7	7,1	16,6	2,1	8,1	0,33	1,54	1,41	0,23	1,34	0,26	0,79	0,11	0,68	0,10	3,17
29/15/2 prim	5,2	6,8	7,1	15,7	2,0	7,6	0,32	1,56	1,40	0,22	1,23	0,26	0,77	0,10	0,68	0,11	3,10
29/15/3	2,5	5,8	5,3	11,4	1,4	5,5	0,26	1,15	1,10	0,17	1,04	0,19	0,55	0,07	0,43	0,06	1,04
29/15/3	2,8	6,0	5,5	11,6	1,4	5,6	0,27	1,20	1,23	0,19	1,04	0,21	0,56	0,08	0,45	0,07	1,29
29/15/3	2,7	5,9	5,6	11,8	1,5	5,7	0,27	1,17	1,11	0,18	1,02	0,21	0,56	0,07	0,46	0,07	1,18
29/15/3	3,4	6,5	6,0	12,5	1,6	6,0	0,29	1,30	1,26	0,19	1,13	0,22	0,63	0,08	0,48	0,07	1,20
29/15/3 prim	2,3	5,7	5,0	10,9	1,3	5,3	0,26	1,13	1,06	0,17	0,97	0,19	0,54	0,07	0,45	0,06	1,08
29/15/3 prim	2,8	5,9	5,3	11,4	1,4	5,6	0,27	1,22	1,19	0,19	1,05	0,21	0,56	0,07	0,46	0,06	1,28
29/15/3 prim	2,7	6,0	5,3	11,4	1,4	5,7	0,27	1,20	1,09	0,18	1,04	0,21	0,56	0,08	0,46	0,07	1,22
29/15/3 prim	3,0	6,1	5,5	11,7	1,5	5,6	0,28	1,21	1,20	0,18	1,06	0,22	0,61	0,07	0,47	0,07	1,18

29/15/4	1,3	4,5	4,2	8,8	1,1	4,3	0,21	0,87	0,82	0,12	0,74	0,14	0,43	0,06	0,36	0,05	1,15
29/15/4	1,7	4,9	4,3	9,1	1,2	4,5	0,22	0,95	0,94	0,14	0,82	0,16	0,47	0,06	0,37	0,05	1,18
29/15/4	1,7	4,9	4,4	9,3	1,2	4,5	0,23	0,95	0,89	0,14	0,86	0,16	0,46	0,06	0,39	0,06	1,25
29/15/4	1,7	4,9	4,3	9,0	1,2	4,4	0,22	0,93	0,91	0,13	0,81	0,16	0,46	0,06	0,38	0,05	1,18
29/15/4 prim	1,1	4,5	4,4	9,0	1,1	4,2	0,19	0,89	0,78	0,12	0,75	0,14	0,42	0,06	0,36	0,05	1,06
29/15/4 prim	1,8	4,8	4,7	9,6	1,2	4,6	0,22	0,95	0,93	0,14	0,84	0,16	0,46	0,06	0,38	0,06	1,15
29/15/4 prim	1,6	4,9	4,7	9,8	1,2	4,6	0,22	0,96	0,88	0,14	0,82	0,16	0,46	0,06	0,37	0,06	1,21
29/15/4 prim	1,5	4,8	4,6	9,4	1,2	4,4	0,22	0,90	0,90	0,13	0,77	0,16	0,43	0,06	0,39	0,06	1,27
29/15/5	2,3	5,7	5,1	11,2	1,4	5,3	0,24	1,12	1,07	0,18	1,07	0,19	0,56	0,07	0,48	0,07	1,28
29/15/5	2,3	5,9	5,0	10,9	1,4	5,2	0,25	1,08	1,19	0,17	0,98	0,19	0,54	0,07	0,47	0,08	1,32
29/15/5	2,4	6,2	5,3	11,7	1,5	5,7	0,26	1,20	1,21	0,18	1,04	0,21	0,61	0,08	0,51	0,08	1,33
29/15/5	2,4	6,3	5,6	12,3	1,5	5,9	0,27	1,22	1,21	0,19	1,11	0,22	0,61	0,08	0,47	0,08	1,41
29/15/5 prim	2,0	6,2	5,5	11,9	1,5	5,7	0,25	1,17	1,12	0,18	1,07	0,20	0,57	0,08	0,50	0,07	1,29
29/15/5 prim	2,2	5,8	5,4	11,9	1,5	5,7	0,26	1,20	1,19	0,18	1,08	0,21	0,58	0,08	0,47	0,07	1,30
29/15/5 prim	2,1	6,0	5,4	11,8	1,5	5,5	0,27	1,15	1,10	0,18	0,98	0,20	0,59	0,08	0,52	0,08	1,41
29/15/5 prim	2,1	6,0	5,5	11,8	1,5	5,6	0,26	1,19	1,16	0,18	1,03	0,20	0,59	0,08	0,51	0,08	1,39
29/15/6	<0,5	1,4	1,4	2,6	<0,5	1,2	0,06	0,25	0,23	<0,05	0,20	<0,05	0,12	<0,05	0,10	<0,05	0,34
29/15/6	<0,5	1,5	1,4	2,5	<0,5	1,2	0,07	0,24	0,28	<0,05	0,21	0,05	0,14	<0,05	0,10	<0,05	0,40
29/15/6	<0,5	1,5	1,5	2,8	<0,5	1,3	0,06	0,29	0,24	<0,05	0,23	<0,05	0,13	<0,05	0,09	<0,05	0,36
29/15/6	<0,5	1,5	1,5	2,7	<0,5	1,3	0,07	0,26	0,29	<0,05	0,22	0,05	0,14	<0,05	0,10	<0,05	0,38
29/15/6 prim	<0,5	1,6	1,6	2,8	<0,5	1,3	0,06	0,27	0,28	<0,05	0,23	<0,05	0,13	<0,05	0,11	<0,05	0,37
29/15/6 prim	<0,5	1,6	1,6	2,8	<0,5	1,3	0,07	0,28	0,27	<0,05	0,24	<0,05	0,14	<0,05	0,10	<0,05	0,34
29/15/6 prim	<0,5	1,5	1,5	2,8	<0,5	1,3	0,06	0,28	0,29	<0,05	0,24	<0,05	0,14	<0,05	0,11	<0,05	0,36
29/15/6 prim	<0,5	1,4	1,4	2,8	<0,5	1,2	0,06	0,28	0,28	<0,05	0,23	0,05	0,13	<0,05	0,11	<0,05	0,33
29/15/7	<0,5	1,7	1,2	2,1	<0,5	1,1	0,05	0,24	0,24	<0,05	0,23	<0,05	0,12	<0,05	0,09	<0,05	0,14
29/15/7	<0,5	1,7	1,3	2,3	<0,5	1,2	0,06	0,27	0,29	<0,05	0,23	<0,05	0,12	<0,05	0,09	<0,05	0,16
29/15/7	<0,5	1,7	1,3	2,3	<0,5	1,2	0,06	0,27	0,26	<0,05	0,23	<0,05	0,13	<0,05	0,10	<0,05	0,17
29/15/7	<0,5	1,6	1,5	2,5	<0,5	1,3	0,07	0,28	0,28	<0,05	0,24	<0,05	0,14	<0,05	0,10	<0,05	0,14
29/15/7 prim	<0,5	1,7	1,2	2,2	<0,5	1,1	0,06	0,24	0,27	<0,05	0,21	<0,05	0,11	<0,05	0,09	<0,05	0,13

29/15/7 prim	<0,5	1,7	1,4	2,3	<0,5	1,2	0,06	0,27	0,29	<0,05	0,24	<0,05	0,13	<0,05	0,09	<0,05	0,15
29/15/7 prim	<0,5	1,7	1,4	2,4	<0,5	1,2	0,06	0,28	0,25	<0,05	0,23	<0,05	0,13	<0,05	0,10	<0,05	0,17
29/15/7 prim	<0,5	1,8	1,3	2,3	<0,5	1,2	0,06	0,26	0,28	<0,05	0,24	<0,05	0,14	<0,05	0,09	<0,05	0,17

29/15/8	<0,5	1,8	1,4	2,3	<0,5	1,2	0,06	0,29	0,26	<0,05	0,24	0,05	0,13	<0,05	0,11	<0,05	0,18
29/15/8	<0,5	1,9	1,4	2,5	<0,5	1,2	0,07	0,27	0,30	<0,05	0,28	<0,05	0,15	<0,05	0,11	<0,05	0,23
29/15/8	<0,5	1,8	1,3	2,4	<0,5	1,2	0,06	0,28	0,27	<0,05	0,25	0,05	0,13	<0,05	0,11	<0,05	0,21
29/15/8	<0,5	1,8	1,4	2,4	<0,5	1,3	0,06	0,30	0,28	<0,05	0,25	0,05	0,14	<0,05	0,12	<0,05	0,24
29/15/8 prim	<0,5	1,8	1,3	2,3	<0,5	1,2	0,06	0,26	0,27	<0,05	0,24	<0,05	0,13	<0,05	0,12	<0,05	0,19
29/15/8 prim	<0,5	1,9	1,4	2,5	<0,5	1,2	0,07	0,29	0,30	<0,05	0,26	0,05	0,14	<0,05	0,11	<0,05	0,28
29/15/8 prim	<0,5	1,9	1,5	2,5	<0,5	1,2	0,07	0,28	0,30	<0,05	0,25	0,05	0,14	<0,05	0,11	<0,05	0,24
29/15/8 prim	<0,5	1,9	1,4	2,5	<0,5	1,2	0,07	0,29	0,27	<0,05	0,27	0,05	0,14	<0,05	0,12	<0,05	0,22

29/15/9	0,5	2,3	1,8	3,6	0,5	1,8	0,09	0,39	0,39	0,06	0,34	0,07	0,19	<0,05	0,15	<0,05	0,34
29/15/9	0,6	2,4	1,9	3,9	0,5	2,0	0,10	0,45	0,45	0,07	0,38	0,08	0,20	<0,05	0,15	<0,05	0,41
29/15/9	0,6	2,5	1,9	4,0	0,5	2,0	0,11	0,45	0,46	0,07	0,38	0,08	0,20	<0,05	0,16	<0,05	0,38
29/15/9	0,6	2,4	1,9	3,9	0,6	2,0	0,10	0,44	0,43	0,06	0,38	0,08	0,20	<0,05	0,16	<0,05	0,38
29/15/9 prim	0,5	2,3	1,8	3,6	0,5	1,9	0,10	0,41	0,40	0,06	0,37	0,07	0,19	<0,05	0,16	<0,05	0,34
29/15/9 prim	0,6	2,4	1,9	4,0	0,5	2,0	0,11	0,46	0,46	0,07	0,40	0,07	0,20	<0,05	0,17	<0,05	0,40
29/15/9 prim	0,5	2,4	1,9	4,0	0,6	2,0	0,11	0,44	0,43	0,07	0,38	0,07	0,21	<0,05	0,16	<0,05	0,41
29/15/9 prim	0,6	2,4	2,0	3,9	0,6	2,0	0,11	0,46	0,45	0,07	0,38	0,08	0,20	<0,05	0,15	<0,05	0,39

29/15/10	<0,5	3,7	1,5	1,9	<0,5	1,3	0,07	0,30	0,38	0,06	0,38	0,08	0,23	<0,05	0,20	<0,05	0,10
29/15/10	<0,5	3,9	1,6	2,0	<0,5	1,5	0,09	0,34	0,44	0,07	0,42	0,09	0,24	<0,05	0,19	<0,05	0,14
29/15/10	<0,5	4,1	1,7	2,1	<0,5	1,5	0,08	0,37	0,43	0,07	0,44	0,09	0,26	<0,05	0,21	<0,05	0,13
29/15/10	<0,5	4,0	1,6	2,1	<0,5	1,5	0,10	0,36	0,40	0,07	0,45	0,10	0,25	<0,05	0,18	<0,05	0,12
29/15/10 prim	<0,5	3,8	1,5	2,0	<0,5	1,4	0,07	0,30	0,38	0,06	0,39	0,08	0,24	<0,05	0,19	<0,05	0,11
29/15/10 prim	<0,5	3,7	1,6	2,0	<0,5	1,4	0,08	0,32	0,38	0,06	0,39	0,08	0,24	<0,05	0,19	<0,05	0,16
29/15/10 prim	<0,5	3,8	1,6	2,1	<0,5	1,5	0,08	0,33	0,44	0,07	0,41	0,09	0,26	<0,05	0,19	<0,05	0,16
29/15/10 prim	<0,5	3,8	1,6	2,1	<0,5	1,5	0,08	0,34	0,42	0,06	0,42	0,09	0,25	<0,05	0,18	<0,05	0,17
